# Bioprinting Decellularized Breast Tissue for the Development of Three-Dimensional Breast Cancer Models

**DOI:** 10.1021/acsami.2c00920

**Published:** 2022-06-23

**Authors:** Barbara Blanco-Fernandez, Sergi Rey-Vinolas, Gülsün Bağcı, Gerard Rubi-Sans, Jorge Otero, Daniel Navajas, Soledad Perez-Amodio, Elisabeth Engel

**Affiliations:** Institute for Bioengineering of Catalonia (IBEC), The Barcelona Institute of Science and Technology (BIST), Baldiri Reixac 10-12, Barcelona 08028, Spain

**Keywords:** decellularization, bioprinting, 3D *in vitro* cancer model, breast tissue

## Abstract

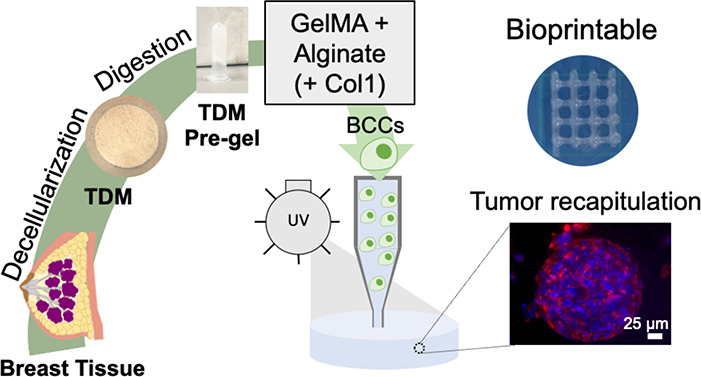

The tumor extracellular matrix (ECM) plays a vital role in tumor progression and drug resistance. Previous studies have shown that breast tissue-derived matrices could be an important biomaterial to recreate the complexity of the tumor ECM. We have developed a method for decellularizing and delipidating a porcine breast tissue (TDM) compatible with hydrogel formation. The addition of gelatin methacrylamide and alginate allows this TDM to be bioprinted by itself with good printability, shape fidelity, and cytocompatibility. Furthermore, this bioink has been tuned to more closely recreate the breast tumor by incorporating collagen type I (Col1). Breast cancer cells (BCCs) proliferate in both TDM bioinks forming cell clusters and spheroids. The addition of Col1 improves the printability of the bioink as well as increases BCC proliferation and reduces doxorubicin sensitivity due to a downregulation of HSP90. TDM bioinks also allow a precise three-dimensional printing of scaffolds containing BCCs and stromal cells and could be used to fabricate artificial tumors. Taken together, we have proven that these novel bioinks are good candidates for biofabricating breast cancer models.

## 1 Introduction

Breast cancer is the most diagnosed cancer in women, yet it is a clinical challenge owing to the complexity of its tumor microenvironment (TME). The breast TME is formed by a heterogeneous population of epithelial breast cancer cells (BCCs), an altered extracellular matrix (ECM), soluble factors (i.e., cytokines and growth factors), and stromal cells, such as adipocytes, immune cells, fibroblasts, or endothelial cells.^1^ Among the components of the breast TME, the ECM is crucial in the tumor outcome, being involved in tumor growth and metastasis, immunosuppression, angiogenesis, or even drug resistance.^1−4^ Indeed, the role of the ECM in tumor progression is still not fully understood due to the difficulty in its evaluation.^2,5,6^

The importance of the tumor ECM has stimulated the creation of new biomaterials that can recapitulate its main physicochemical, biological, and mechanical properties. Of these, proteinaceous hydrogels have been widely used to develop 3D *in vitro* cancer models^7^ as they can mimic important ECM features such as cell adhesion sites, biodegradation sequences, viscoelasticity, mechanical properties, and architecture.^8^ In breast cancer modeling, hydrogels made of collagen type 1 (Col1) and gelatin methacrylamide (GelMA)^1^ have been widely used to recreate the stiffness of the ECM.^7^ However, the absence of tumor ECM components in these biomaterials can limit their application when studying the role of the ECM in cancer physiopathology. Hydrogels made of a native ECM have also been used to recreate the breast cancer ECM, with Matrigel being the gold standard in cancer modeling.^9−11^ However, the high variability between batches and undefined composition of Matrigel can affect the model’s reproducibility. Decellularized tissues and organ-derived matrices (TDMs) have recently been explored to recreate the tumor ECM.^12^ TDMs consist of complex protein mixtures combined with other molecules, which retain important biological cues of the native tissue.^13^ TDM hydrogels and scaffolds have shown their extraordinary bioactive properties, such as cell proliferation and behavior,^12,14^ enabling the development of more physiologically relevant cancer models. Decellularized breast and adipose tissues can mimic the ECM as a whole, but they are still relatively underexplored.^15−18^ Indeed, Ruud and coworkers reported that BCCs have a different metabolic profile, invasiveness, and morphology to native cancer cells when cultured in a decellularized tissue material compared to Matrigel or Col1 hydrogels.^19^ Therefore, it has been shown that decellularized breast tumors could recapitulate the native breast tumor.^20^ This evidence suggests that biomaterials based on breast TDMs could be a better candidate to mimic the breast tumor ECM in breast cancer 3D *in vitro* models.

Within the TME, there are complex interactions between cells and the ECM, cancer and stromal cells are arranged hierarchically, and their organization evolves during the tumor progression.^21^ 3D bioprinting can assist in the replication of this cellular organization. More biomimetic, anatomically relevant, and complex models have been achieved using this technology, recreating in this way the physiology and functionality of tissues.^22,23^ In breast cancer, 3D bioprinting has allowed the development of tumor models with precise control over BCCs and stromal cell location,^24^ enabling the deposition of different cell types in different locations and with different bioinks. For example, models of the interface between the bone and breast tumors were created by 3D bioprinting to study the metastases to these tissues.^25^ Also, hydrogels consisting of a core of BCCs and an outer layer of adipose-derived mesenchymal stem cells (hAMSCs) made by 3D bioprinting have shown that MSCs increase the resistance of BCCs against oncology drugs.^26^ Despite the advances in this field, there is still an urgent need to increase the availability of tumor biomimetic bioinks to study the crosstalk between the tumor ECM, stromal cells, and BCCs. In recent years, new efforts have been made to develop bioinks using TDMs for tissue engineering applications and cancer modeling.^27^ However, these biomaterials do not possess suitable mechanical properties to be bioprinted without the use of sacrificial materials or a supporting secondary structure,^14^ and the stiffness of TDM hydrogels cannot recreate the tumor stiffness. To allow the TDM bioprinting by itself, researchers have incorporated rheological modifiers into the bioink or chemically functionalized the TDM to incorporate photopolymerizable groups.^28,29^

In this work, we present an approach for bioprinting cell-laden hydrogels made of a breast TDM, without the requirement of any sacrificial material, which has never been reported before. We propose the incorporation of alginate and GelMA to a porcine breast TDM for the fabrication of bioinks with suitable mechanical properties for 3D bioprinting and hydrogels with appropriate stiffness to recreate the tumor. The addition of GelMA into the bioinks enables the printability of the TDM with suitable shape fidelity, whereas the incorporation of alginate ensures an appropriate Young’s modulus and physical integrity. This novel bioink provides the mechanical and biological cues necessary for the fabrication of breast cancer models. To show the unique properties of this bioink and its versatility in tumor modeling, we evaluate the effect of Col1, which is overexpressed in breast tumors, in tumor progression and drug effectivity.

## 2 Experimental Section

### 2.1 Materials

Alginate (ref. 71238), bovine serum albumin (BSA), calcein AM, DNase I, calcium chloride (CaCl_2_), deuterium oxide, doxorubicin, fetal bovine serum (FBS), gelatin from porcine skin type A, hydrochloric acid (HCl), isopropanol, methacrylic anhydride (MA), *N*-(2-hydroxyethyl)piperazine-*N*′-(2-ethanesulfonic acid) (HEPES), oil red O, paraformaldehyde, pepsin, phalloidin–tetramethylrhodamine B isothiocyanate, sodium deoxycholate (SDC), sodium hydroxide (NaOH), Triton X-100, 3-((3-cholamidopropyl)dimethylammonium)-1-propanesulfonate (CHAPS), and 4′,6-diamidino-2-phenylindole (DAPI) were acquired from Sigma Aldrich. A Tissue-Tek O.C.T. Compound and Irgacure 2959 were purchased from Sakura Finetek and BASF, respectively. Advanced Dulbecco’s modified Eagle’s medium (advanced DMEM), Dulbecco’s phosphate-buffered saline (DPBS) 10×, l-glutamine, penicillin–streptomycin, phosphate-buffered saline (PBS), and propidium iodide (PI) were purchased from Thermo Fisher Scientific. Goat serum, an anti-E-cadherin antibody (ab201499), and Alexa Fluor 488 goat antirabbit IgG H&L (ab150077) were purchased from Abcam.

### 2.2 Preparation of TDMs

Breast tissues from 3–6-month-old female pigs were purchased from the Comparative Medicine and Bioimage Centre of Catalonia (Spain) after the approval by their ethical committee and immediately frozen and stored at −80 °C to avoid ECM degradation. Frozen tissues were thawed at 4 °C and washed several times with PBS before the mammary glands were surgically harvested. Then, mammary tissues were sliced into 1–2 cm^3^ pieces, homogenized in ice-cold deionized ddH_2_O in a domestic blender, and spun (3000*g*, 5 min, RT). The precipitated tissue was collected, and this process was repeated four times to guarantee maximum fat removal. Then, the breast tissues were decellularized at 4 °C under magnetic stirring. Tissues were incubated in 4% SDC for 24 h and 8 mM CHAPS for 24 h (with a change of each solution after 12 h). After each decellularization of solution, the tissues were washed with deionized ddH_2_O for 1 h. Next, tissues were incubated with DNase I (100 U/mL, 2 h, RT), washed with ddH_2_O (20 min, three times), incubated with isopropanol for 24 h (change after 12 h, 4 °C), washed with ddH_2_O (20 min, three times), freeze-dried, and stored at −80 °C. TDMs were then milled at 5 min at 10 CPS in liquid N_2_ using a freezer mill (6775 SPEX SamplePrep) and stored again at −80 °C. Rat and mouse mammary glands were kindly provided by Prof. del Río (Institute for Bioengineering of Catalonia, Spain) and the Institute for Advanced Chemistry of Catalonia (Spain), respectively. Rat and mouse breast tissues were subjected to the same decellularization protocol, but these tissues were not blended nor spun due to their small size.

### 2.3 TDM Characterization

The content of collagen, GAGs, and cells remaining in the TDM powder was evaluated. The remaining DNA was determined using a Quant-iT PicoGreen dsDNA assay kit (Thermo Fisher Scientific). The total glycosaminoglycan (GAG) content was measured using a Glycosaminoglycan Assay Blyscan kit (Biocolor) following the manufacturer’s instructions. The collagen content was quantified by a hydroxyproline method (Supporting Information). Part of the TDMs before cryomilling was embedded in paraffin or a Tissue-Tek O.C.T. Compound and sectioned. Hematoxylin and eosin staining (H&E) was performed to ensure the complete removal of the cells. Collagens were visualized with a picrosirius red stain kit (Abcam), and the remaining fat in the TDMs was determined by oil red O staining.

### 2.4 Cell Culture

MCF-7 (HTB-22, ATCC) and hAMSCs were cultured in advanced DMEM supplemented with 10% FBS, 1% penicillin–streptomycin, and 1% l-glutamine. hAMSCs were obtained from adipose tissue samples from an anonymous donor after his consent (Delfos Hospital).^30^

### 2.5 TDM Hydrogel Preparation

Powder TDMs were resuspended at 5% in a solution of pepsin at 0.5% in 0.1 N HCl and stirred at room temperature for 16 h. Then, the solution was stored at 4 °C for 1 day and used for the hydrogel preparation. Briefly, the TDM solution was neutralized with 1 M NaOH, and DPBS 10× was added to guarantee a suitable osmolarity for cell culture. To obtain the desired concentrations (10, 20, or 30 mg/mL), PBS or cell culture media was added in appropriate amounts (Table 1).

**Table 1:** Composition of the Bioinks Used^a^

components	TDM	GelMA	alginate	Col1	Irgacure	cross-linking
T1	1%					37 °C
T2	2%					37 °C
T3	3%					37 °C
T2_A0.5	2%		0.5%			37 °C + CaCl_2_
T2_A1	2%		1%			37 °C + CaCl_2_
T2_A2	2%		2%			37 °C + CaCl_2_
T2_G4_A0.5	2%	4%	0.5%		0.1%	37 °C + CaCl_2_ + UV
T2_G2.5_A0.5 (TGA)	2%	2.5%	0.5%		0.1%	37 °C + CaCl_2_ + UV
T2_G2.5_A0.5_Col1 (TGAC)	2%	2.5%	0.5%	0.15%	0.1%	37 °C + CaCl_2_ + UV

a NaOH was added to each bioink to neutralize the pH, being adjusted individually. DPBS 10× was added to guarantee the cell-friendly osmolarity of the bioink and was calculated according to the volume of TDM, Col1, and NaOH used. PBS or cell media was added to reach 100% in each bioink.

### 2.6 TDM Bioink Preparation

GelMA was fabricated as previously reported with some modifications.^31^ Gelatin was dissolved in PBS at 10% at 40 °C. MA was added dropwise under stirring and left to react for 1 h to have a final concentration in the reaction mixture of 4.8% v/v. Then, the reaction mixture was dialyzed against ddH_2_O at 40 °C for 5 days (MWCO of 12.4 kDa) and freeze-dried. The degree of substitution (DS) was calculated by ^1^H NMR in deuterium oxide,^32,33^ by calculating the areas of the methylene protons of lysine (3.1–3.2 ppm) according to the following equation:
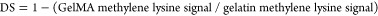
1

Each value was normalized by the area of aromatic amino acids (7.2–7.5 ppm). The resulting DS was 53%.

Col1 was extracted from rat tails as previously published.^34^ The total protein content of Col1 was quantified by microBCA (Thermo Fisher Scientific) using Col1 as a standard (OptiCol Rat Collagen Type I, no. MS18, Cell Guidance Systems).

The TDM bioink composition is described in Table 1, using the format T*X*_G*X*_A*X*_Col1, where “*X*” is the concentration in % (w/v) of TDM (T), GelMA (G), or alginate (A), and Col1 was used at 0.15% in all formulations. When a letter in the code is missing, this indicates its absence from the bioink, and in order to simplify, the selected bioinks were named TGA (T2_G2.5_A0.5) and TGAC (T2_G2.5_A0.5_Col1), and they were prepared as follows. First, TDM digested as specified in Section 2.3 was placed in an ice bath, and the pH of the solution was neutralized with 1 M NaOH. Then, enough DPBS 10× was added to the solution to have a final concentration 1× to ensure a cell-friendly osmolarity. Second, solutions containing GelMA, Irgacure 2959, and alginate were prepared in PBS. Finally, both solutions were mixed, placed in bioplotter syringes, and stored at 4 °C before their use. In the case of cell-laden bioinks, the cell suspension was added lastly. The TDM bioink with Col1 was prepared in the same manner, by diluting the Col1 solution into the TDM.

### 2.7 Bioprinting

TDM bioinks were printed with a 3D bioplotter (RegenHU, 3D Discovery). Bioinks were extruded through a dispensing tip with an internal diameter of 0.51 mm (Nordson) at temperatures ranging from 8 to 20 °C, pressures of 0.25–0.5 bar, and speeds of 3–5 mm/s. The hydrogels were then cross-linked by UV light (365 nm), by incubating them for 2 min with 0.58% CaCl_2_ in 10 mM HEPES and by placing them in a cell incubator for 30 min at 37 °C. When bioinks were forming drops instead or a continuous linear filament, a bed of 4 mL of Pluronic at 23% at 37 °C was used. Pluronic beds were removed by several washes with cold PBS.

### 2.8 Printability and Shape Fidelity of TDM Bioinks

TGA and TGAC bioinks were bioprinted at 8 °C and 0.5 bar and a speed of 4 mm/s. The formation of a filament or a drop after extrusion was verified visually. Twelve filaments of 14 mm were bioprinted, and images were acquired with a stereomicroscope (Leica). Their diameter and length were calculated using ImageJ software (National Institutes of Health). The filament diameter was determined at 30 different locations. The spreading ratio was calculated by dividing the diameter of the printed filament by the internal diameter of the needle.^35^ Filament fusion tests were performed with one layer of the bioink. The scaffold showed the architecture described in Figure 5A. The printability and the diffusion rate were calculated as previously described.^36^
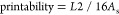
2
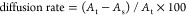
3where *A*_t_ is the theoretical pore area, *A*_s_ the pore area of the scaffold, and *L* the perimeter of the pore. The pore’s morphology was determined visually by fabricating scaffolds with theoretical pores of 3 × 3 mm (area after subtracting the thinness of the bioink, 0.51 mm of 6.2 mm^2^). The angle shape of filaments with an L shape was assessed visually by bioprinting filaments as described in Figure 5A.

### 2.9 Mechanical Testing

The rheological properties of the bioinks were measured with a rheometer (dynamic mechanical analyzer MCR 702, Anton Paar) using a cone plate of 40 mm diameter (CP40-1, no. 2627, Anton Peer) with a gap of 0.078 mm. Temperature sweeps were performed from 4 to 37 °C at an increasing rate of 4 °C/min at a shear rate of 1 Hz.^37^ Amplitude sweeps at 1 Hz and at a temperature of 8 (for TGA and TGAC) or 37 °C (for T2) were carried out. Flow curves at variable shear rates were also obtained at 8 °C. In all cases, TDM bioinks obtained from different digestions were used (*N* = 3). The Young’s modulus of TGA and TGAC hydrogels was measured with a Zwick column with a load cell of 5 N (Zwick, Roell, Germany) using hydrogels of 400 μL prepared in 48-well plates. Compression tests were carried out at a speed of 50% deformation/min (*N* = 3). Hydrogels made of only a TDM, alginate, GelMA, and their combinations (see Table S1) were also prepared under the same conditions to determine the effect of each component on the hydrogel’s stiffness.

### 2.10 Bioink Merging during Bioprinting

To study if TGA and TGAC bioinks enable the 3D printing of several cell types with a precise location and without the blending of the layers for an appropriate recapitulation of breast tumors, colored bioinks were prepared with food coloring. A scaffold consisting of a core for recreating the tumor site and an outer layer to mimic the stromal layer in breast tumors was designed (Figure 6A). TGA and TGAC bioinks were prepared as described before, and then, a drop of food coloring was added. Bioinks with no food coloring were used for the inner core (tumor core), and bioinks stained in red were used for the outer layer (stromal layer). Cell-laden scaffolds with an MCF-7 core and an hAMSC outer layer were also bioprinted (Figure 6A) to ensure that there was no cell merging when bioprinting. hAMSCs and MCF-7 were marked with Vybrant DiO and DiD, respectively, according to the manufacturer’s instructions (Thermo Fisher Scientific) and then resuspended in the bioink *(*hAMSCs, 1 × 10^6^ cells/mL; MCF-7, 1.5 × 10^6^ cells/mL). Cell-laden scaffolds were cultured in the same media as cancer cells and kept in culture for only 1 day. Then, hydrogels were fixed with 4% PFA and visualized in a Thunder Imager 3D live cell microscope (Leica Microsystems).

### 2.11 Bioprinting Cell-Laden Bioinks

MCF-7 cells were resuspended in the bioinks at a concentration of 1.5 × 10^6^ cells/mL unless otherwise stated and bioprinted under the same conditions as described before. The scaffold architecture is described in Figure 7A. Cell media was replaced every other day in all cell-laden hydrogels, and they were kept for 14 days in culture.

### 2.12 Cellular Staining

Cell survival on the bioprinted and nonbioprinted hydrogels was evaluated with live/dead staining using calcein AM/PI. Cell-laden hydrogels at different time points were washed twice with PBS at 37 °C and incubated with 2 μM calcein AM and 4 μM PI in PBS for 20 min. The cell viability was calculated with a 3D object counter in FIJI software.^38^ For immunofluorescence images, cell-laden hydrogels were washed twice with PBS, fixed with paraformaldehyde (20 min, RT), permeabilized with 0.1% Triton X-100 (5 min, RT), blocked with 10% goat serum in 3% BSA in PBS (1 h, RT), incubated with an anti-E-cadherin antibody (1:250, overnight, 4 °C), incubated with goat antirabbit IgG H&L (1:1000, 1 h, RT), and incubated with phalloidin–tetramethylrhodamine B isothiocyanate (45 min, RT) and DAPI (10 min, RT). Three washes of 3% BSA in PBS were done between each step. To study the cellular morphology in hydrogels, cells were stained only with phalloidin and DAPI, using the same procedure. Hydrogels were visualized with a Thunder Imager 3D live cell microscope (Leica Microsystems). Nonbioprinted hydrogels made of Col1 at 4 mg/mL encapsulating MCF-7 at 1.5 × 10^6^ cells/mL were used as controls.

### 2.13 Cell Proliferation and Drug Response

Cellular proliferation in the bioprinted hydrogels was measured with alamarBlue (Thermo Fisher Scientific). At different time points, cell media was replaced by alamarBlue at 10% in cell media. After 1 h of incubation, fluorescence was measured (560/590 nm). Four replicas per condition were used. A calibration curve of MCF-7 cells was also obtained to determine the cell density. For drug response studies, cell-laden bioprinted hydrogels were cultured for 14 days to enable MCF-7 growth. Then, hydrogels were incubated with doxorubicin at different concentrations for 2 days, and the cell viability was determined by alamarBlue as specified before. Hydrogels were washed with PBS twice before a solution of 10% alamarBlue in the cell media was added. Then, hydrogels were incubated for 1 h at 37 °C, and the fluorescence was measured. Nontreated bioprinted hydrogels were used as controls. IC_50_ values were calculated using GraphPad Prism 8.0 software (GraphPad Software). 2D experiments and Col1 hydrogels at 4 mg/mL (20 μL) were run in parallel as controls. In order to study if the cell density had any effect on the drug response, nonbioprinted TGA, TGAC, and Col1 cell-laden hydrogels (20 μL) containing 1.5 or 3 × 10^6^ cells/mL were cultured and treated under the same conditions as bioprinted hydrogels.

### 2.14 Real-Time Quantitative Polymerase Chain Reaction (RT-qPCR)

In order to analyze the expression of tumoral markers before and after the treatment with doxorubicin, TGA, TGAC, and Col1 cell-laden hydrogels were cultured for 14 days followed by a 48 h drug treatment (0 or 0.1 μM, 16 days in total). Cell-laden hydrogels were washed with PBS and stored at −80 °C in 350 μL of RLT lysis buffer containing 1% β-mercaptoethanol until RNA isolation. For 2D samples, cells were cultured for 9 days with a 48 h doxorubicin treatment (0 or 0.1 μM). On the day of RNA isolation, samples were sonicated on ice (50% amplitude, 3 cycles of 30 s, with each cycle followed by 30 s of vortexing). Then, samples were freeze–thawed three times. After centrifugation at +4 °C (5 min, 13,000*g*), the supernatant was transferred to a gDNA Eliminator spin column, and RNA isolation was performed using an RNeasy Plus Mini Kit (Qiagen, no. 74134) following the manufacturer’s instruction. The RNA concentration was measured using a Nanodrop instrument (ND-1000, NanoDrop). cDNA synthesis from RNA was performed by using an RT2 First Strand Kit (Qiagen, no. 330404). An appropriate amount of cDNA was mixed with an RT2 SYBR Green ROX qPCR master mix (Qiagen, no. 330524) and a primer mix. Human β-actin was acquired from Qiagen (ACTB, PPH00073G-200), whereas the rest of the primers were purchased from Sigma Aldrich (sequences of primers listed on Table S1). RT-qPCRs were run in a StepOnePlus system (Applied Biosystems) under the following conditions: 1 cycle of 10 min at 95 °C followed by 40 cycles for 15 s at 95 °C and 1 min at 60 °C, and the following melting curves were run. Experiments were performed in triplicate with three technical replicates per sample. The fold change in the gene expression was calculated using the 2^–ΔΔCt^ method, using β-actin as a housekeeping gene.

### 2.15 Statistical Analysis

GraphPad Prism 8.0 software (GraphPad Software) was used to run the statistical analysis. *t*-Tests and one-way or two-way ANOVAs, whenever appropriate, were used to study whether there were statistical differences between conditions, with *p* values below 0.05 being considered statistically different. All data points presented on charts are the mean values ± standard deviation (*n* = 3, unless specified).

## 3 Results and Discussion

The role of the ECM in tumor progression has motivated the creation of novel biomimetic biomaterials that recreate the tumor ECM complexity. In this regard, a native breast tissue has already shown its suitability in the creation of relevant cancer models.^15,39^ The similarities between the human and pig genome, availability, and large size of the pig mammary glands have prompted the use of this type of breast tissue for the preparation of a biomimetic ECM bioink.^40^ To the best of our knowledge, this is the first time that decellularized breast tissue bioinks mimicking the mechanical and biochemical characteristics of the breast ECM have been developed.

### 3.1 Decellularization and Delipidation of Breast Tissues

**Scheme 1:**
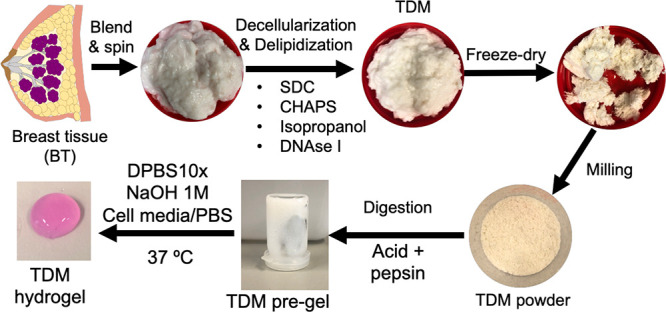
Schematic Illustration Showing the Workflow of the TDM Hydrogel Fabrication

The decellularization and delipidation of breast tissues with minimal damage and loss of the ECM are critical when preparing hydrogels from TDMs.^41^ The whole porcine breast tissues were decellularized and delipidated as described in the Experimental Section and are summarized in Scheme 1. In our protocol, we combined the use of two mild detergents, SDC and CHAPS, to decellularize the tissue, isopropanol and physical treatment (blending) to remove the lipids of the tissue, and the incubation with DNase I to ensure the removal of any remaining DNA.

**Figure 1:**
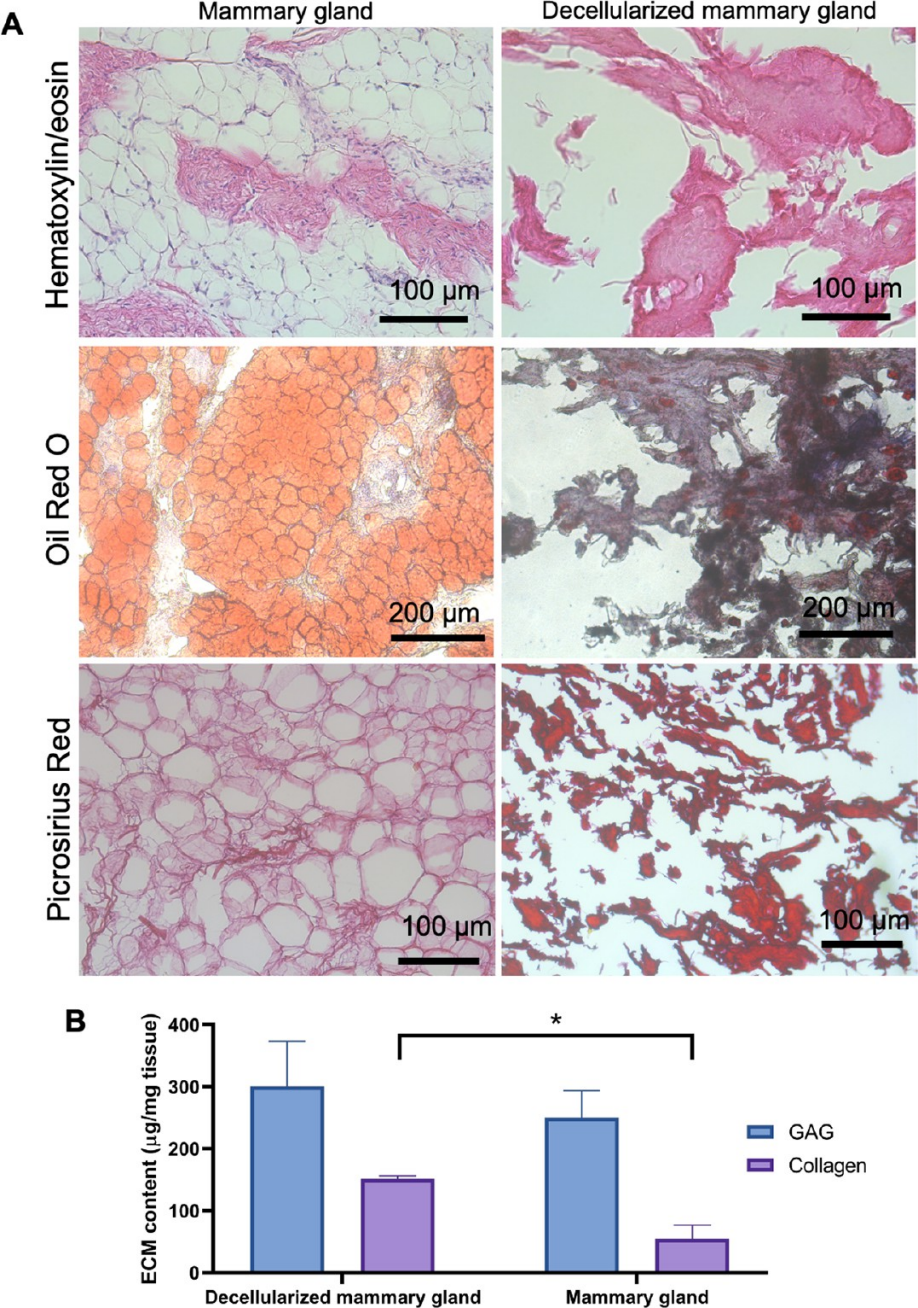
(A) Histological comparison of the native porcine breast tissue (left) and the decellularized breast tissue (TDM, right). Hematoxylin and eosin staining of tissue sections (scale bar of 100 μm), oil red O staining (scale bar of 200 μm), and picrosirius red staining (scale bar of 100 μm). (B) Total collagen and GAG contents in the porcine breast tissue and the decellularized breast tissue (TDM).

The efficiency of decellularization was assessed through the quantification of the remaining DNA in the biomaterial, being below 5 ng/mg dried ECM (4.53 ± 2.10 ng/mg). TDMs were also stained with H&E to examine the presence of any remaining cellular components after the decellularization process. As shown in Figure 1A, cellular nuclei were not observed. The absence of the nuclear material (Figure 1A) and DNA values below 50 ng/mg dried ECM indicated a suitable and efficient decellularization process.^14,41^ In addition, the efficiency of the delipidation was assessed through oil red O staining of tissue sections. Figure 1A clearly shows that the decellularization and the physical delipidation combined with the isopropanol treatment provoked a high lipid reduction when compared with the native breast tissue. However, some lipid drops were still visible, something which has been previously seen with other decellularization processes of breast or adipose tissues.^17^

### 3.2 TDM Biochemical Characterization and Jellification

Since mammary glands consist of lipids, collagens, and GAGs,^42^ the quantity of these last two macromolecules in TDMs and native tissues was evaluated (Figure 1B). We found a high content of collagen and GAG after the harsh decellularization and delipidation processes, suggesting that TDM could recreate the human breast ECM. We observed a statistically significant increase of total collagens in TDMs when compared with the native tissue, which could be explained by the removal of the lipids as well as the cellular components from the breast tissues. Picrosirius staining of tissue sections also confirmed the high content of collagen in TDMs. Also, the GAG content was slightly higher in the case of the TDMs, but this was not statistically significant.

**Figure 2:**
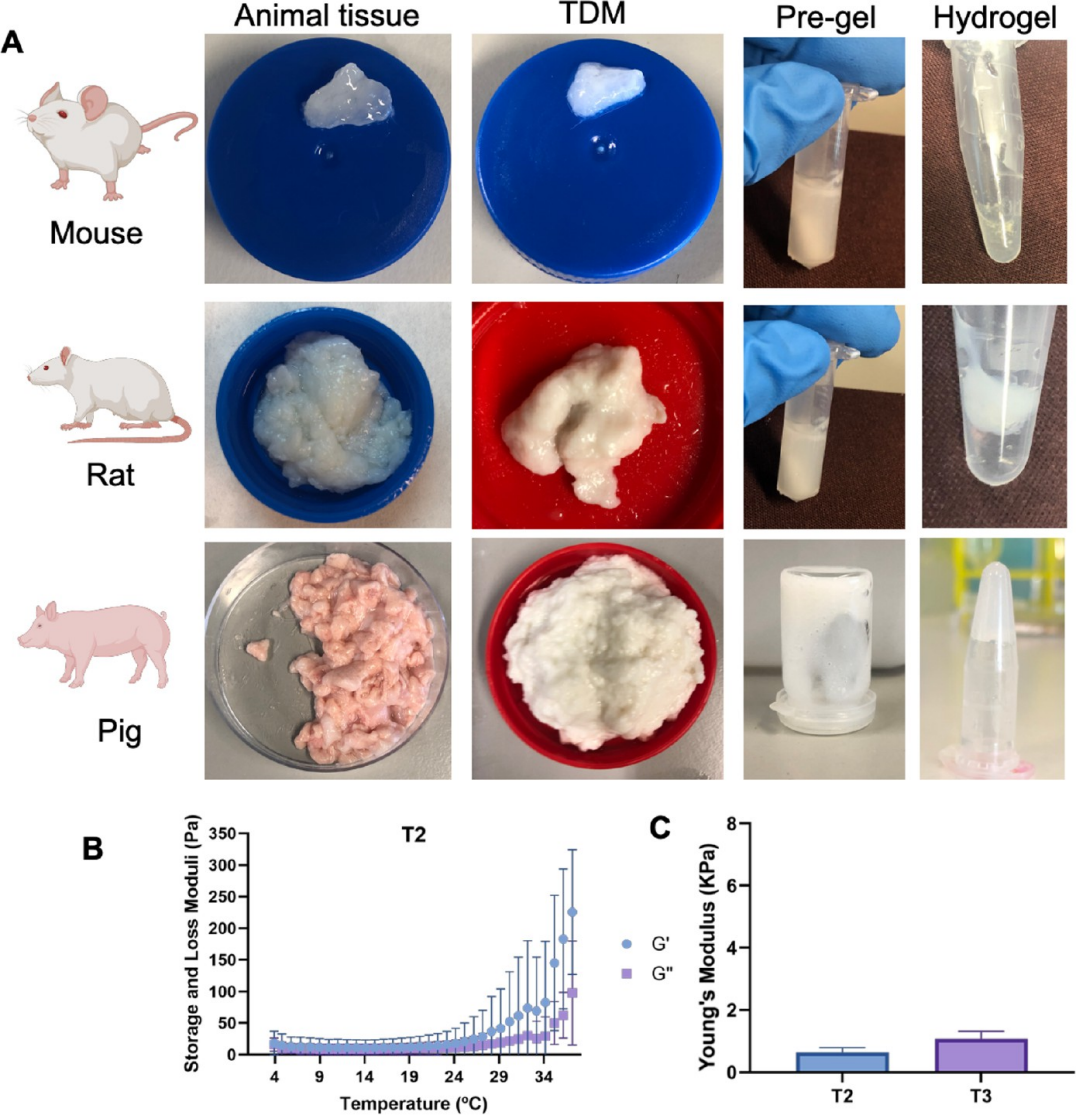
(A) Hydrogel formation from TDMs of various sources. Mouse, rat, and porcine breast tissues (first column) were decellularized (second column), lyophilized, cryomilled, and enzymatically digested (third column), and pre-gel solutions were used to prepare TDM hydrogels (fourth column). (B) Rheological properties of T2 at different temperatures. (C) Young’s moduli of T2 and T3 hydrogels.

Then, the capability of the porcine TDM to gel was visually tested (Figure 2A). TDMs were cryomilled and digested with pepsin in an acid solution. TDM pre-gel solutions (T1, T2, and T3) were formed by neutralizing the pH and correcting osmolarity to ensure its biocompatibility, keeping the solution at 4 °C to avoid the cross-linking of the ECM. Next, hydrogels were cross-linked by incubating them at 37 °C (Scheme 1 and Figure 2A). The inversion of the vial after the incubation showed that the biomaterial was able to gel in a thermally dependent manner, mainly caused by the presence of collagen. This result was confirmed by rheometry, showing an increase in the storage modulus above 30 °C (Figure 2B). The Young’s modulus of TDM hydrogels was also determined. T1 hydrogels were too fragile to be handled, and their Young’s modulus could not be measured. In contrast, T2 and T3 hydrogels were easier to handle with moduli of 0.64 ± 0.16 kPa for T2 and 1.08 ± 0.24 kPa for T3 (Figure 2C). The increase in the TDM concentration in the hydrogel allowed an increase in the Young’s modulus, but it was not statistically significant (*t*-test, *p* = 0.057), and none of the hydrogels could recreate the stiffness reported in human breast tumors,^43,44^ which has been already seen in hydrogels made of TDMs of other origins.^29^

Mouse and rat breast tissues were also evaluated to check whether this protocol was translatable to other animal tissues. For this purpose, tissues were subjected to the same decellularization process, but in this case, the blending step could not be performed due to the small size of the tissues (Figure 2A). Unfortunately, rat breast tissues could only partially gel, whereas mouse breast tissues could not form any gel, and only the porcine breast tissue rendered TDMs able to cross-link and form hydrogels (Figure 2A). We hypothesize that this phenomenon could be due to the high lipid content remaining in the rat and, especially, in the mice breast TDMs. Also, the differences in the ECM composition between species, which has been already reported for other tissues,^45^ might interfere with the jellification of the biomaterial, suggesting that the decellularization process should be adjusted to each individual tissue species.

### 3.3 TDM Hydrogels Support Cell Proliferation

**Figure 3:**
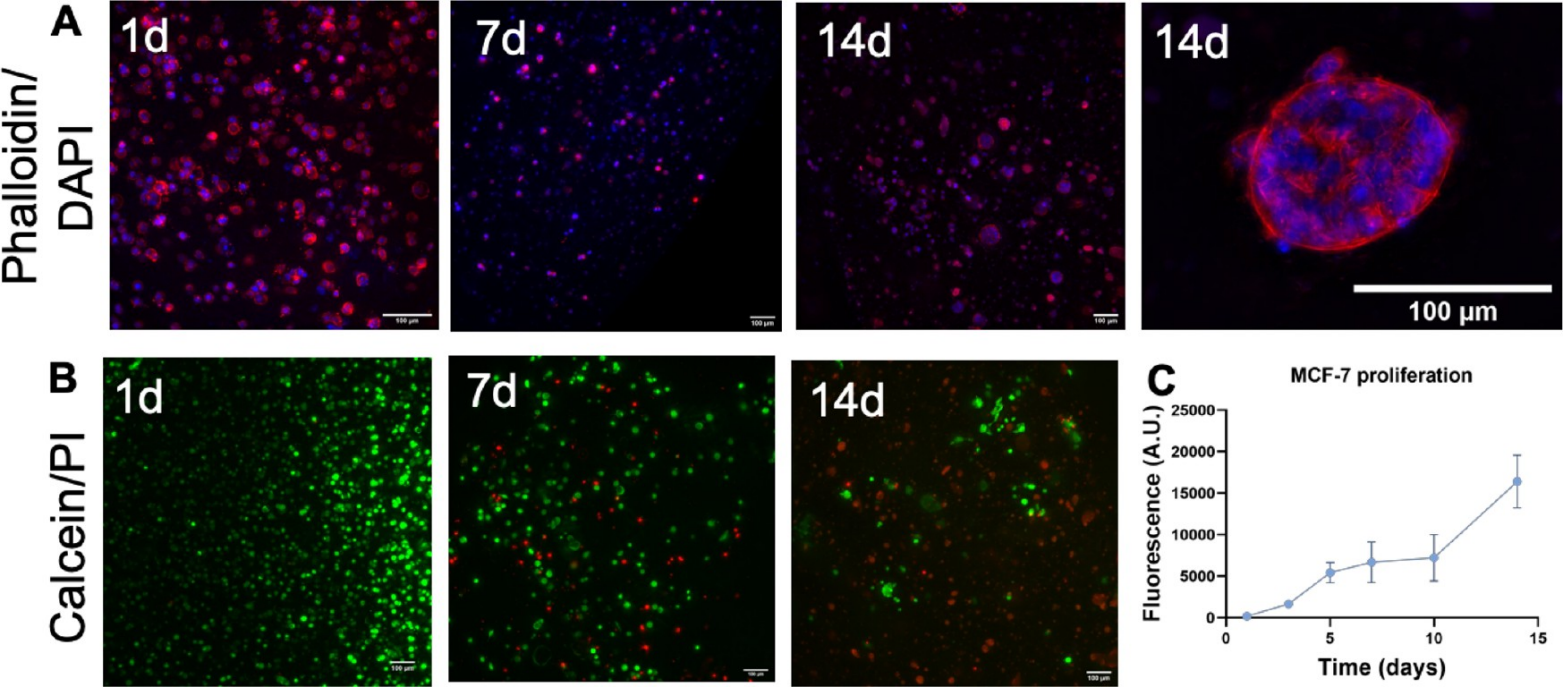
MCF-7 viability, proliferation, and organization in TDM hydrogels at 2%. (A) Cellular organization over time (cell cytoskeletons stained with phalloidin (red) and nuclei stained with DAPI (blue), scale bar of 100 μm). (B) MCF-7 viability over time (viable cells stained with calcein AM in green and dead cells stained with PI in red, scale bar of 100 μm). (C) Cellular proliferation over time by alamarBlue.

To study the cellular behavior and cytocompatibility of TDM hydrogels, MCF-7 cells were dispersed in a T2 pre-gel solution, and then, hydrogels were prepared by incubating them at 37 °C. The cellular distribution, cytocompatibility, and proliferation were evaluated with phalloidin/DAPI staining, vital staining, and alamarBlue (which has been reported to be suitable for measuring the proliferation of cancer cells in proteinaceous hydrogels).^15^ MCF-7 cells were homogeneously distributed in the hydrogels, and at day 14, BCCs formed cell aggregates (Figure 3A). TDM hydrogels were cytocompatible on day one (Figure 3B). However, at day 7 and, especially, on day 14, some cell death was observed. Surprisingly, cellular proliferation was increased up to day 14 (Figure 3C). This result might be explained by the formation of some spherical cell aggregates observed at day 14 (Figure 3A). These results confirmed the cytocompatibility of the biomaterial and the ability of MCF-7 cells to form cell spheroids in TDM hydrogels.

### 3.4 The Incorporation of Alginate and GelMA into the TDM Pre-gel Solution Allows TDM Bioprinting

Then, we tested the bioprintability potential of TDM hydrogels. The first step in testing the printability of a bioink is the formation of a linear filament when extruded through the nozzle.^46^ T1, T2, and T3 pre-gel solutions were extruded through the nozzle at temperatures ranging from 8 to 20 °C. However, no filament could be formed to allow the bioprinting of the biomaterial by itself. Amplitude sweeps confirmed the low storage modulus of the TDM, which explains the unsuitability of the biomaterial to be bioprinted by itself (Figure S6D). The use of a sacrificial material was also tested to check if it enabled the TDM bioprinting. A Pluronic F127 bed at 23% was used (Figure S1) as a bed, as it is easily removed with cold PBS washing. Bioinks with TDM concentrations of 20 (T2) and 30 mg/mL (T3) were the only ones successfully bioprinted in the bedding. These TDMs were jellified at 37 °C for 30 min, and then, the bedding was removed through several washes with cold PBS to solubilize the Pluronic (Figure S1). None of the scaffolds made with T2 maintained their shape after the removal of the bedding, whereas some scaffolds with T3 did. As the increase in TDM concentration did not allow the bioprinting of the biomaterial with good shape fidelity neither suitable stiffness to recreate the breast tumor, the addition of rheological modifiers was studied. In any case, the conditions of temperature and pressure needed to 3D print TDMs did not affect the cellular viability of MCF-7 (Figure S2).

**Figure 4:**
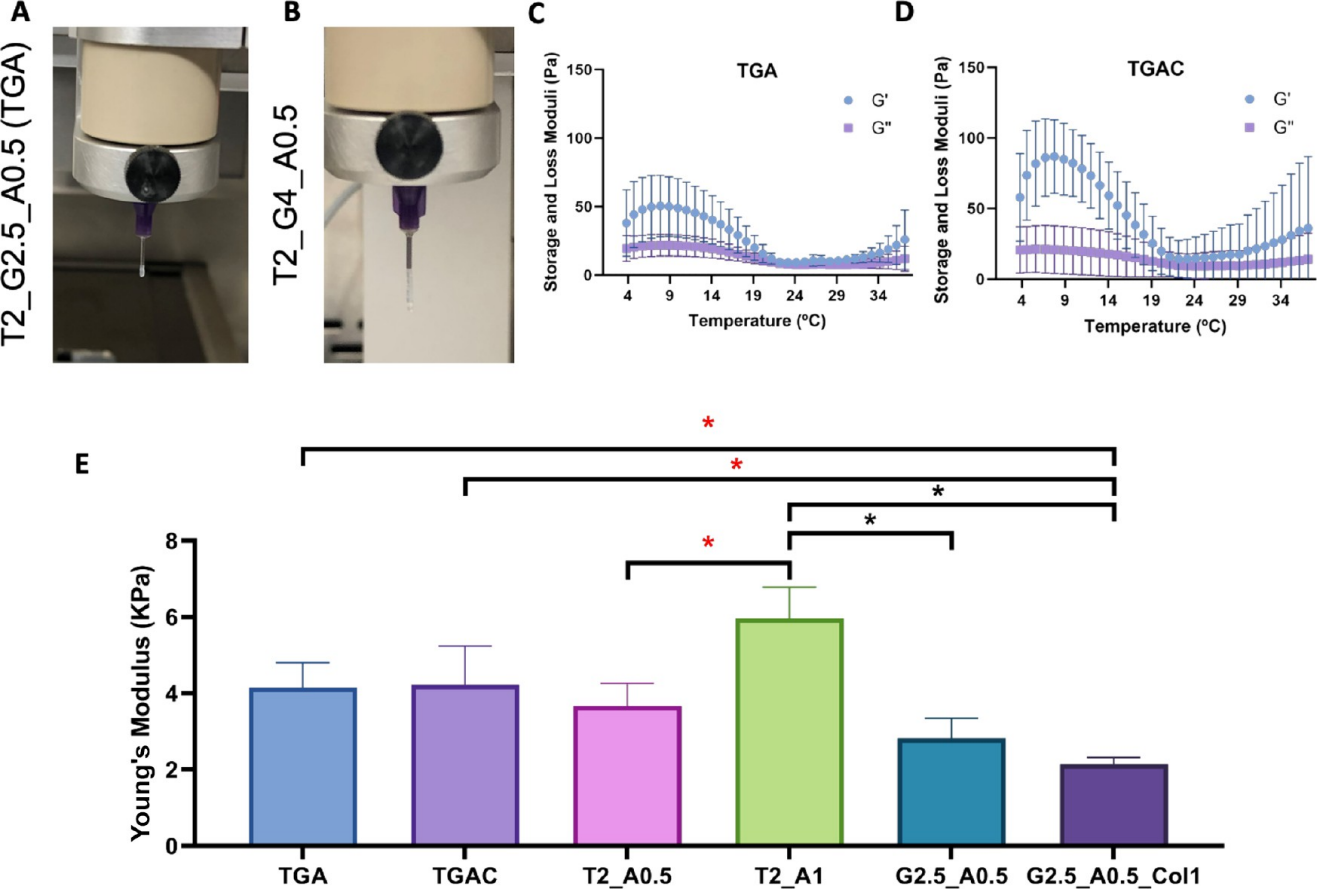
(A,B) Formation of linear and continuous filaments of TDM bioinks containing 20 mg/mL TDM, 0.5% alginate, and 2.5 (A, TGA bioink) or 4% (B, T2_G4_A0.5) GelMA. (C,D) Temperature sweeps of TGA (C) and TGAC (D) bioinks. (E) Young’s moduli of TGA, TGAC, T2_A0.5, T2_A1, G2.5_A0.5, and G2.5_A0.5_Col1 hydrogels.

In order to improve the TDM bioink printability as well as increase the Young’s modulus of the hydrogels, we first explored the addition of alginate to the TDM pre-gel solutions at different concentrations (0.5, 1, and 2%) (Figure 4 and Figures S3 and S4). We selected alginate as a rheological modifier, as it has allowed the bioprinting of collagen bioinks before, which is one of the components of the TDM.^47^ The incorporation of alginate did not alter the TDM thermal mechanical properties (Figure S3A), but it increased the hydrogels’ stiffness (Figure 4C) to more closely recreate the breast TME. However, the concentrations tested were still not enough to allow the formation of a continuous linear filament through the nozzle, requiring again the use of a sacrificial material (Figure S3B). Nevertheless, MCF-7 cells were viable up to 14 days in culture in these hydrogels (Figure S4). Interestingly, BCCs were not able to form cell aggregates at alginate concentrations above 1%, implying that the addition of alginate as a rheological modifier was not sufficient to allow the TDM bioprinting by itself at the concentrations needed to allow cell interactions.

In order to avoid the use of a sacrificial material, a combination of GelMA (2.5–4%) and alginate (0.5%) was added. We decided to use GelMA as it is a commercially available bioink that has been widely used for bioprinting tissues^48^ and is also recently shown to be beneficial for bioprinting other decellularized materials.^29^ We also decided to incorporate alginate to ensure suitable stiffness to recreate the tumor, as alginate has been previously added into other bioinks with suitable properties for cell culture.^29^ In this case, all the conditions tested enabled the formation of a filament at temperatures below 15 °C (Figure 4A,B), and the lowest concentration of GelMA (2.5%) was used for further studies. Temperature sweeps showed that the addition of GelMA at 2.5% provoked a modification in the rheological behavior of the TDM, with the rheological properties being similar to other GelMA bioinks.^49^ In addition, the highest storage modulus was achieved at 8 °C (Figure 4C), and therefore, we decided to bioprint the bioink at 8 °C. As the addition of GelMA at 2.5% into the hydrogels makes them more fragile, hindering the hydrogel manipulation, alginate at 0.5% was also incorporated in the bioink to give them more stability as well as to allow hydrogel stiffness comparable to breast tumors, being reported between 4 and 5.7 kPa (Figure 4E).^43,44^

To study the effect of each component on the final hydrogels’ stiffness, the Young’s modulus of each component as well as their combinations were measured. GelMA at 2.5% had a modulus comparable to TDM hydrogels (Figure S5), whereas alginate at 0.5% had a higher modulus (2.96 ± 1.29 kPa, Figure S5), proving that the addition of alginate into the bioink will have a greater impact on the hydrogels’ stiffness. We decided to use the lowest concentration of alginate tested (0.5%), as it allowed the formation of higher numbers of spheroids (Figure S4) at the same time as it rendered hydrogels with the Young’s modulus in the range of breast tumors. The TDM also had a positive impact on the hydrogel’s stiffness, as its absence reduced its Young’s modulus (Figure 4E). Therefore, we selected T2_G2.5_A0.5 (TGA) for further studies.

### 3.5 TDM Bioinks Allow the Incorporation of ECM Proteins Improving Their Bioprintability

The ECM plays an active role in breast cancer progression, metastasis, and drug resistances.^42^ During breast tumor development, the ECM experiences alterations in composition, structure, and mechanical properties, in comparison with healthy tissues;^42^ for instance, the production of fibrillar collagens, fibronectin, laminin, and proteoglycans is increased.^42^ Among ECM proteins, Col1 is overexpressed in breast tumors and is involved in the proliferation, survival, and metastasis of BCCs.^50^ Since the porcine TDM used for the bioink fabrication has a nontumoral origin, as it is obtained from healthy female pigs, we decided to modify its composition by including Col1 in the TGA bioink (TGAC). This ECM protein is produced by cancer-associated fibroblasts, and it is involved in tumor progression, metastasis, and the stiffening of the tumor.^42^ We decided to tune the bioink only with one molecule overexpressed in breast tumors to analyze whether its addition may promote breast cancer ECM proliferation and survival, avoiding changes in the printability of the biomaterial and the stiffness. Therefore, the addition of Col1 in GelMA hydrogels has shown to increase BCC invasion.^51^ A concentration of 0.15 mg/mL of Col1 was added to the bioink as it was able to modulate the activity of BCCs.^52^

First, we evaluate the printability and mechanical and rheological properties of the TDM bioink, and then, we assess whether the addition of Col1 had any impact on the bioink properties. The incorporation of Col1 in the TDM bioink did not modify the thermal behavior of the bioink obtained in the temperature sweeps, being also the highest storage modulus at 8 °C (Figure 4D). Therefore, TGAC was also bioprinted at this temperature to ensure the optimal shape fidelity. In addition, Col1 did not alter the bioink printability, forming a linear and continuous filament when extruded at 8 °C.

**Figure 5:**
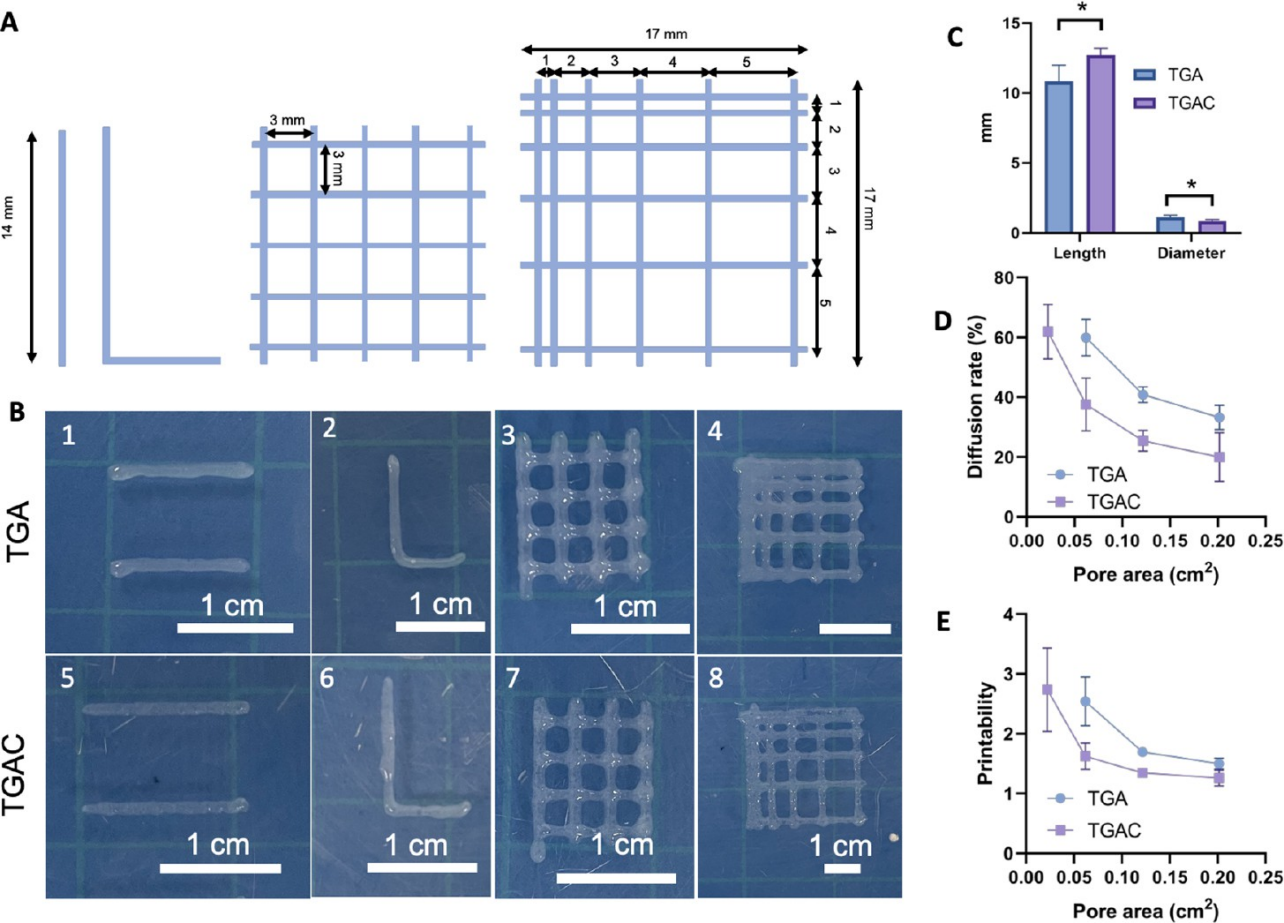
(A) Design of the bioprinted scaffolds to study the printability and shape fidelity. (B) Bioprinted scaffolds to determine the printability and shape fidelity of the bioinks. (1,5) Filaments of 14 mm in length, (2,6) filaments with an L shape (3,7) scaffolds with pores of 3 × 3 mm (4,8), and scaffolds for the filament fusion test. (C) Length and diameter of bioprinted filaments (theoretical sizes: 14 mm length, 0.51 mm diameter). (D) Diffusion rate of both bioinks. (E) Printability of both bioinks.

To evaluate the bioink printability and shape fidelity, we first studied the shape fidelity of single lines by quantifying the filament’s diameter and spreading ratio. TGA and TGAC bioinks showed a higher diameter than the internal diameter of the dispensing tip (0.51 mm) and a shorter length than the designed filament (14 mm, Figure 5A, B(1,5), and C). We also evaluated the spreading ratio for both bioinks, with these values being similar to previously reported spreading rates for GelMA at 10%.^35^ In this case, the TGAC bioink has a statistically significant lower spreading ratio (1.69 ± 0.18) than the TGA bioink (2.21 ± 0.28) (*t*-test, *p* < 0.0001). Nevertheless, both values are accepted to be low enough to ensure a precise bioprinting.^35^ Next, the roundness/sharpness of the angle in filaments with an L shape (90° angle) was evaluated. The addition of Col1 impacted the corner of the filament, being sharper when Col1 was incorporated into the bioink (Figure 5A,B(2,6)). The morphology of pores in bioprinted scaffolds was also assessed by fabricating constructs with one layer and 3 mm × 3 mm pores (Figure 5A,B(3,7)). No visual differences were observed between both bioinks. The fusion of the filaments was also calculated by measuring the diffusion rate and printability, as described in the methodology section, using scaffolds with the architecture specified in Figure 5A,B(4,8). Both bioinks showed a decrease in diffusion and an increase in printability at larger pore sizes (Figure 5D,E). Interestingly, the addition of Col1 improved the diffusion rate as well as the printability at small pore sizes (<0.09cm^2^, *p* < 0.001). All these results indicate that the addition of a low concentration of Col1 (0.15%) had a positive impact on the printability and shape fidelity of the scaffold. The higher storage modulus of the TGAC bioink compared to the TGA bioink allows the 3D printing of constructs with better precision.

TGA and TGAC bioinks have shear-thinning properties, which are crucial for bioprinting through extrusion (Figure S6A).^29^ Amplitude sweeps showed that the presence of Col1 in the bioinks enabled a 1.5-fold increase in the storage modulus at 8 °C, confirming the better properties for bioprinting of the TGAC bioink (Figure S6B,C). In both cases, the storage moduli were higher in the TGA and TGAC bioinks than in only the TDM (Figure S6D). The mechanical properties of TGAC hydrogels were also measured. TGA and TGAC hydrogels showed similar Young’s moduli of approximately 4 kPa (TGA, 4.15 ± 0.66 kPa; TGAC, 4.22 ± 1.02 kPa, Figure 4E), proving that the addition of Col1 at this concentration did not modify the stiffness of the hydrogels and that their stiffness can recreate the stiffness of breast tumors.

**Figure 6:**
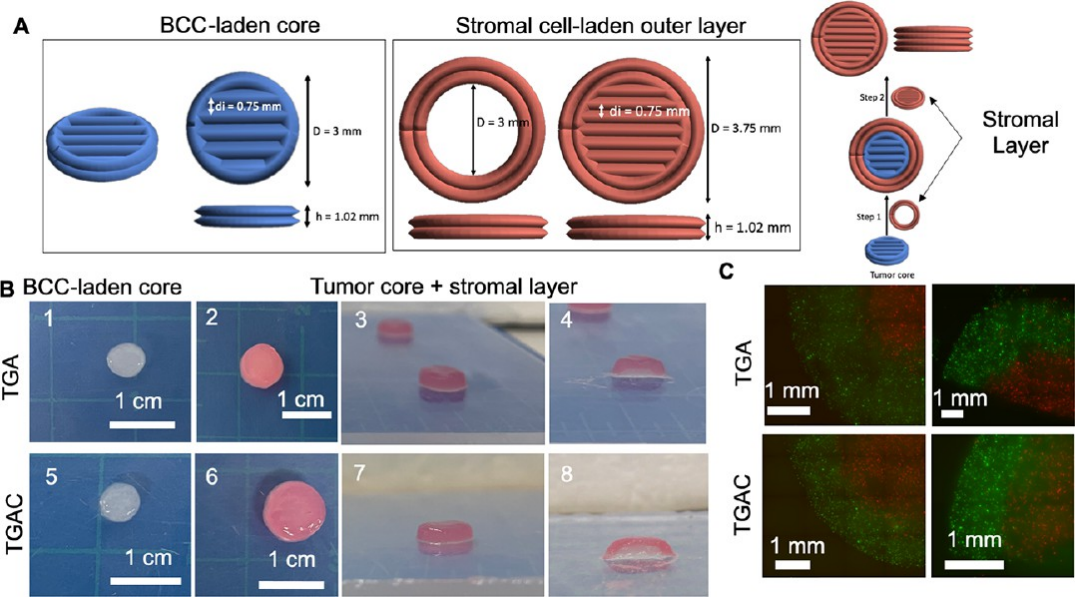
MCF-7- and hAMSC-laden bioprinted scaffolds. (A) Architecture of the bioprinted scaffolds with BCC- and stromal-cell-laden hydrogels (diameter, *D*; interline distance, di; height, *h*). (B) Bioprinted scaffolds (without cells) with a white core (1,5) recreating the tumor and an outer layer in pink (2,3,6,7) mimicking the stromal layer and cut in half (4,8). (C) Bioprinted hydrogels consisting of a core of MCF-7 (in red) and an outer layer of hAMSCs (in green). On the left is the hydrogel view from the bottom of the hydrogel, and on the right is the hydrogel view from the middle after slicing it in half.

The crosstalk of BCCs and stromal cells plays a vital role in tumor progression as well as drug resistances, and therefore, it needs to be recreated in 3D *in vitro* models.^53,54^ In this way, 3D bioprinting could assist in the creation of more physiological relevant models, by allowing the control of the location of BCCs and stromal cells in a precise and accurate manner.^24^ To determine whether TDM bioinks could be used to bioprint cancer and stromal cells, with a precise location of both cell types, a scaffold consisting of a core that recreates the BCC environment and an outer layer of stromal cells (human adipose mesenchymal stem cells, hAMSCs) was bioprinted (Figure 6A). First, a scaffold with two different colors of bioinks was fabricated to verify whether it was possible to bioprint BCCs and stromal cell-laden bioinks without their merging. A core of two layers was made to recreate the tumor environment (white bioink), and an outer layer with four layers (red bioink) was chosen to recreate the stromal environment. Both bioinks could be used to fabricate this type of scaffold without any fusion of the bioinks in the interface (Figure 6B). To ensure that this interface between stromal cells and BCCs was maintained after the bioprinting, hAMSCs and MCF-7 cells were marked with a cell tracker, and images of the bioprinted scaffolds were taken. Both cell types were localized in the bioprinted area, with no evident merging of hAMSCs or MCF-7 (Figure 6C). Overall, these findings suggest that TDM bioinks could be used for the bioprinting of more complex cancer models, where a precise location of cancer and stromal cells is needed.

### 3.6 TDM Bioprinted Scaffolds Can Be Used to Study the Role of the ECM in BCC Progression and Drug Resistance

**Figure 7:**
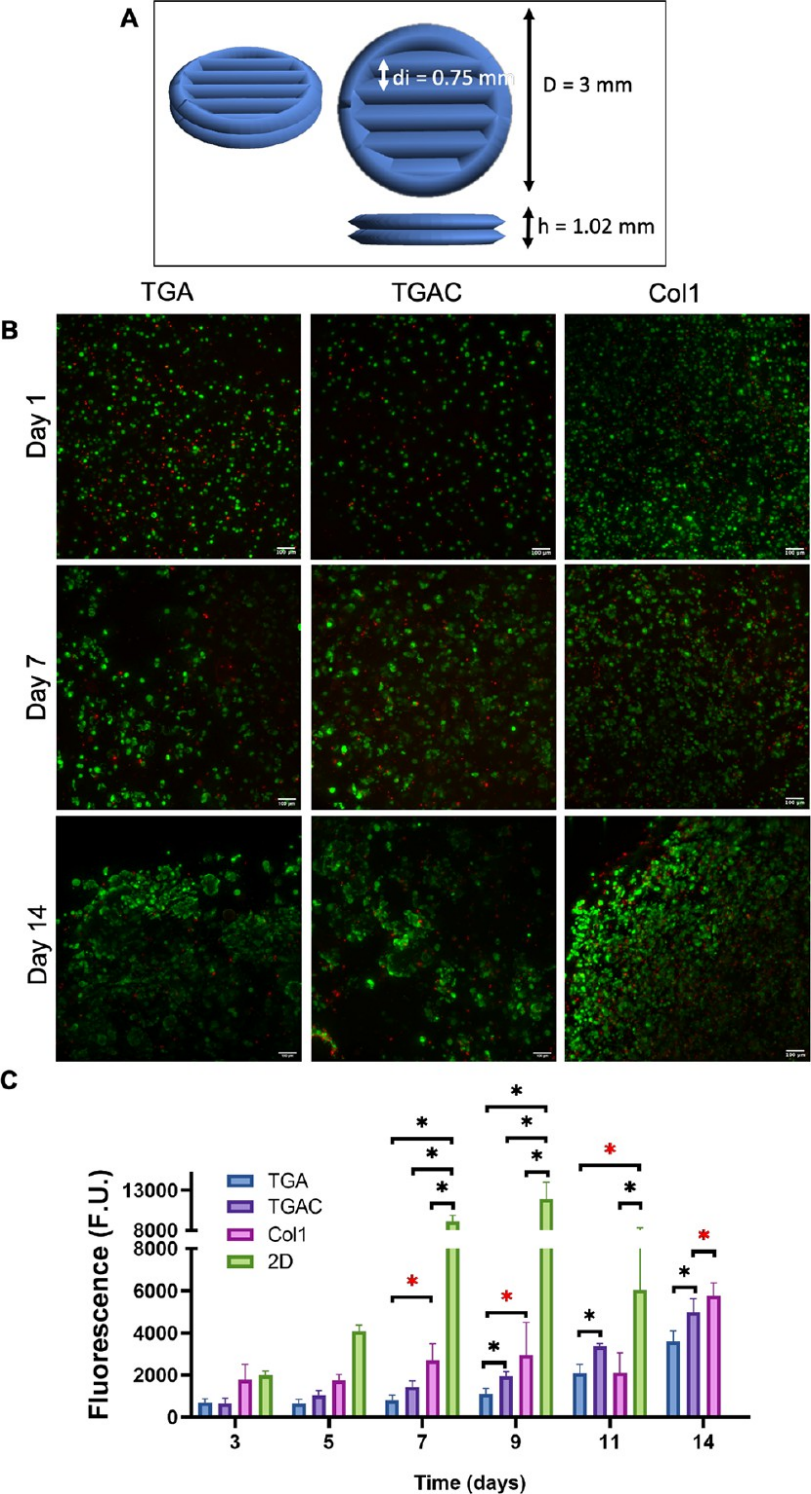
MCF-7-laden bioprinted scaffolds. (A) Architecture of the scaffolds (diameter, *D*; interline distance, di; height, *h*). (B) Cell viability after 1, 7, and 14 days in culture (in green are cells stained with calcein AM (alive), and in red are cells dyed with PI (dead)). (C) MCF-7 proliferation by alamarBlue.

Finally, we tested whether the presence of Col1 in the developed bioinks had an impact on BCC proliferation and drug efficacy outcomes. MCF-7 cells were resuspended in TGA and TGAC bioinks, and cylinder scaffolds recreating the tumor core shown in Figure 7A were bioprinted. Hydrogels were maintained for 14 days in culture with media replacement every other day. Vital staining and the cell proliferation by alamarBlue were carried out at different time points. It is important to note that the mechanical properties were sufficient to allow hydrogel handling. Calcein AM/PI staining at day 1 showed that MCF-7 cells were homogeneously distributed in both hydrogels, indicating that they were successfully resuspended in the bioink, and no sedimentation was observed during the hydrogel cross-linking (Figure 7B). Bioprinted BCC-laden hydrogels showed good cytocompatibility up to 14 days in culture (Figure 7B and Table S3), comparable to Col1 hydrogels, with no visible hydrogel disintegration. As Col1 may influence BCC proliferation, an assay using alamarBlue was performed at different time points (Figure 7C) and compared to cells growing in 2D and Col1 hydrogels. In both bioinks and Col1 hydrogels, the cell proliferation was higher in cells growing in 2D than in hydrogels after 7 days in culture; however after 11 days in 2D, cells started to detach from the wells. Cells growing in the bioprinted hydrogels showed an increase in proliferation in the presence of Col1 at 0.15% at all time points, but this was only statistically significant from day 9 until the end of the culture period. Indeed, TGAC hydrogels showed cell proliferation and density values comparable to Col1 hydrogels (Figure 7C and Figure S6). This finding suggests that the presence of Col1 has a positive impact on cell proliferation. Furthermore, as there are no differences in the Young’s moduli between both types of hydrogels, the differences in cell proliferation observed may be due to the addition of Col1 and not to changes in the stiffness of the hydrogels.

**Figure 8:**
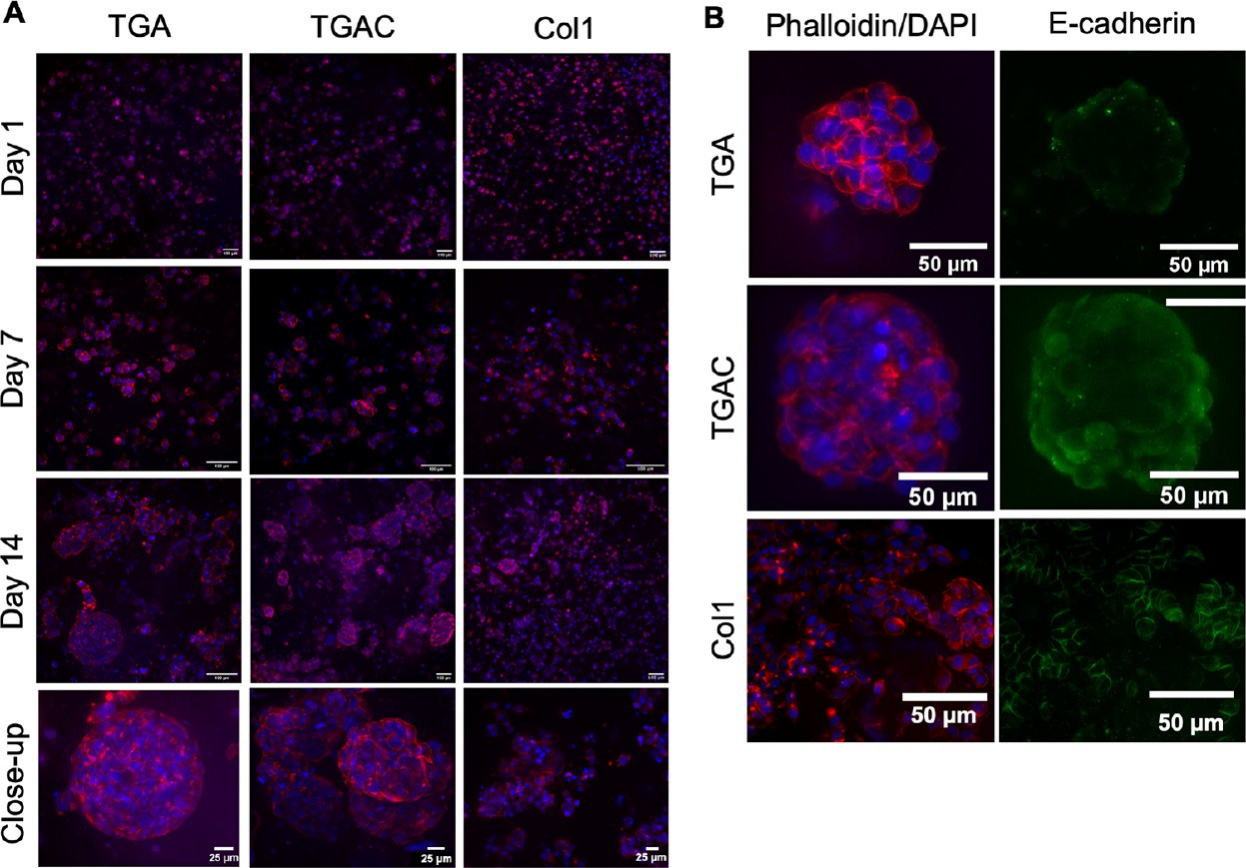
MCF-7-laden bioprinted scaffolds. (A) MCF-7 stained with phalloidin–rhodamine (red) and DAPI (blue) at day 1, 7, and 14. (B) Expression of E-cadherin (green) in MCF-7 clusters after 14 days in culture.

Next, BCC distribution in the hydrogels was studied. Phalloidin/DAPI staining showed that cells were homogeneously and individually distributed in the bioprinted hydrogel on day 1 (Figure 8A). On day 7, MCF-7 cells formed small cell clusters, and only on day 14, proper cell spheroids and cell clusters were observed. When Col1 was incorporated in the TDM bioink, no differences between cell aggregates were observed (Figure 8A). Interestingly, differences were observed when comparing these hydrogels with Col1. MCF-7 cells were able to form cell aggregates colonizing the full hydrogel, which might be explained by the lower stiffness of Col1 hydrogels (<1 kPa).^55^

The expression of E-cadherin was also evaluated. This adhesion molecule is present in diverse breast cancer types, and its lower expression is linked with tumor progression and metastasis.^56^ Interestingly, this adhesion molecule was not highly expressed, being only present in some cell aggregates and especially in hydrogels containing Col1 (Figure 8B), which might be explained by the presence of Col1, as this marker was highly expressed in Col1 hydrogels. This finding agrees with previous results using decellularized adipose tissue scaffolds. Dunne and coworkers showed a lower expression of this adhesion molecule in TDM scaffolds when compared with Matrigel and 2D cultures of MCF-7.^15^

**Figure 9:**
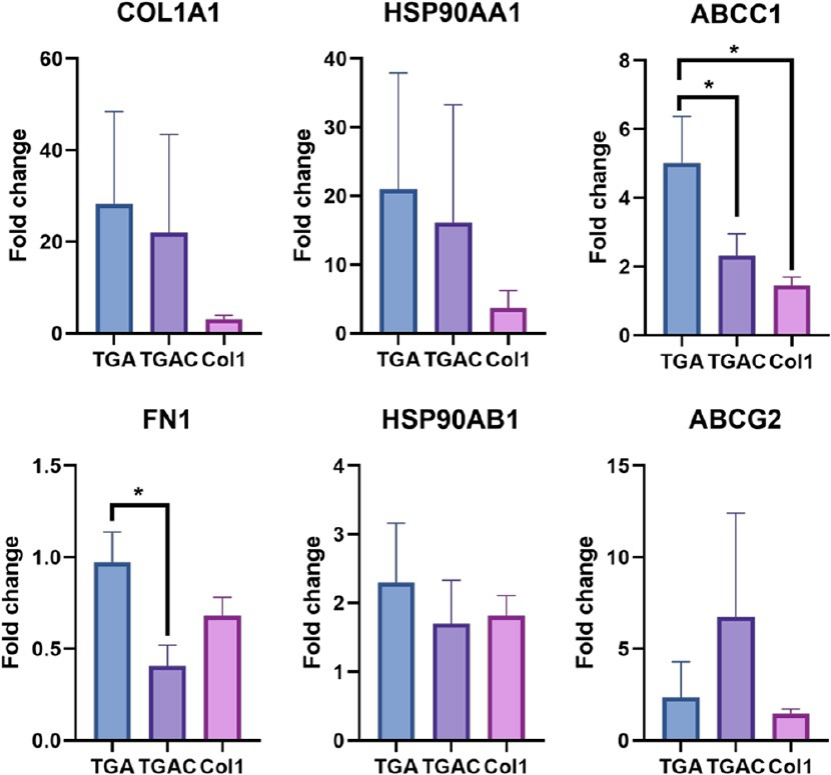
COL1A1, FN1, HSP90AA1, HSP90AB1, ABCC1, and ABCG2 expressions in MCF-7 cells cultured in TGA, TGAC, or Col1 for 14 days. 2^–ΔΔCt^ values were calculated with the ΔCts from 2D cultures.

We further explored if the presence of Col1 in the bioinks has a positive impact on the expression of malignant tumor markers, such as Col1, fibronectin, heat shock proteins of 90 kDa (HSP90), and calcium and potassium ion channels (Figure 9 and Figure S8). We first explored the expression of Col1 (COL1A1) and fibronectin (FN1), as both are hallmarks of breast cancer and are linked to poor prognosis and metastasis.^42,57−59^ Cells grown in TGA, TGAC, and Col1 exhibited an upregulation in the expression of COL1A1 in comparison to 2D cultures, suggesting a malignant transformation in all hydrogels (Figure 9). Nevertheless, there were no statistical differences between conditions, implying that the addition of Col1 at 0.15% was not sufficient to have a synergic effect between the TDM and Col1 in the upregulated COL1A1. Conversely, there was a downregulation of FN1 in the cell-laden hydrogels, especially in the case of TGAC, which implies that the absence of Col1 is beneficial to maintaining the fibronectin expression (Figure 9). We also evaluated the expression of two chaperone HSP90s, Hsp90alpha (HSP90AA1) and HSP90beta (HSP90AB1), due to their involvement in many breast cancer pathways, metastasis, angiogenesis, and antiapoptotic activity, and their upregulation is related to poor prognosis.^60,61^ We also found a gene upregulation for both proteins in comparison with 2D cultures, but no differences among conditions were detected (Figure 9). Ca^2+^ signaling has also shown its involvement in tumor progression,^62^ with the T-type voltage-gated calcium channels such as Cav3.1 (CACNA1G) being involved in its regulation in numerous cancer cells. In MCF-7, the overexpression of Cav3.1 has been linked with a reduction in the cell proliferation.^63^ Both bioprinted hydrogels showed no expression of CACNA1G (Ct values above 35) in contrast to 2D cultures (Figure S8), suggesting that cells growing in TGA and TGAC had a closer phenotype to breast cancer cells, where this gene is low expressed.^64^ We also decided to study the expression of Kv1.1 protein (KCNA1) as it has been reported to be expressed in MCF-7 cells and has also been involved in the progression and the malignancy grade of breast tumors.^65^ Again, no expression of KCNA1 (Ct values above 35, Figure S8) was found, whereas it was expressed in cells growing in 2D, indicating that cells growing in TGA and TGAC can recreate a reduction in the expression of this specific channel occurring *in vivo*.^66^ We also evaluated if TGA and TGAC bioinks might promote the expression of multidrug resistance proteins (MDR), by measuring the expression of multidrug resistance-associated protein 1 (MRP1, encoded by ABCC1) and breast cancer resistance protein (BCRP, encoded by ABCG2) (Figure 9). Both MDR proteins were more highly expressed in the cell-laden hydrogels than in 2D, but only TGA showed a higher upregulation of ABCC1. Altogether, the addition of Col1 into the TDM bioink does not cause a synergistic effect in the expression of drug resistance and tumor malignancy markers. Moreover, the TGA bioink’s higher expression of MRP1 and fibronectin indicates that the addition of Col1 might not improve the biological properties of the TDM.

**Figure 10:**
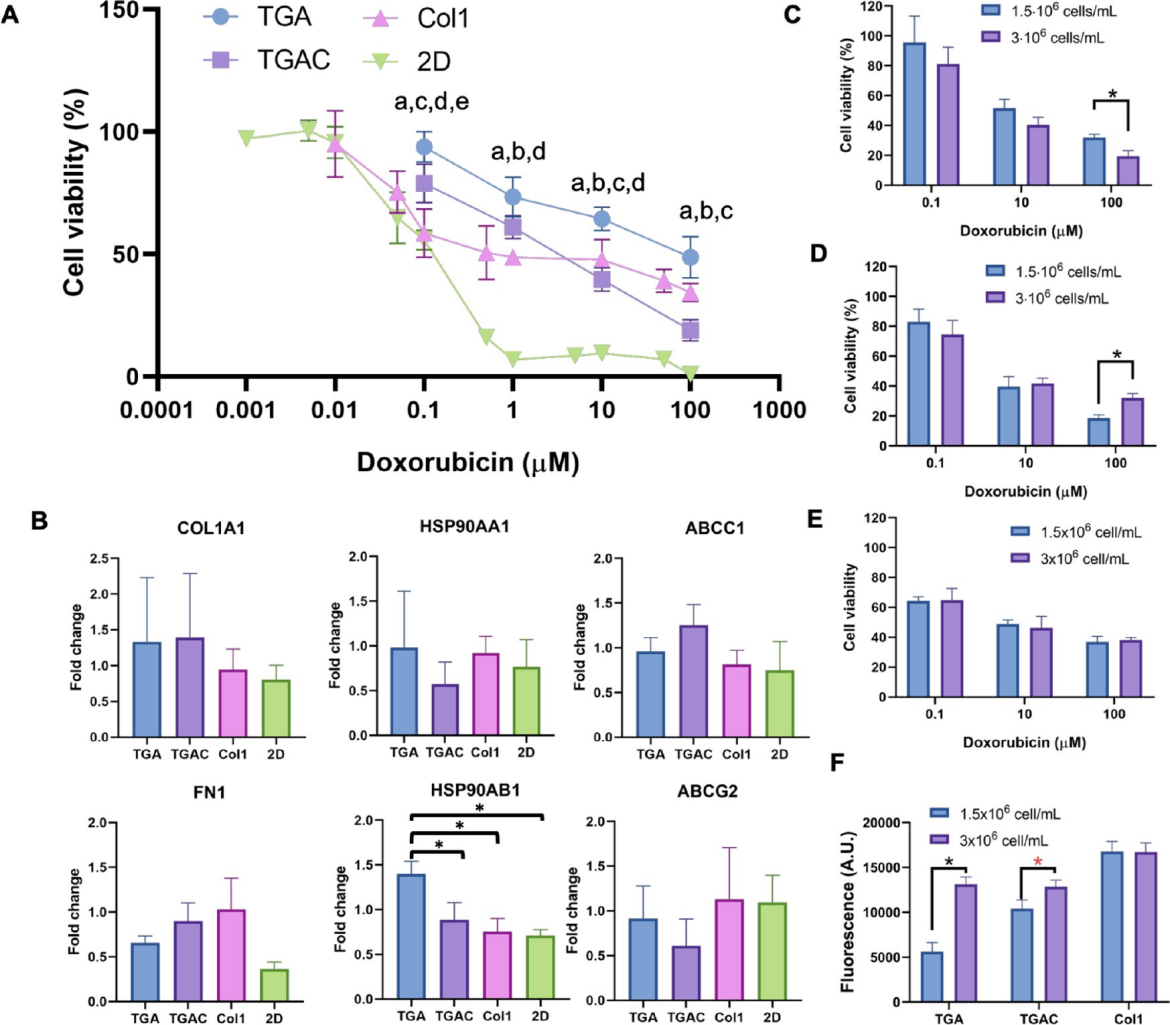
(A) MCF-7 viability after incubating the cell-laden hydrogels with doxorubicin for 48 h (a: *p* < 0.001 for TGA or TGAC vs the 2D control, b: *p* < 0.001 for Col1 vs the 2D control, c: *p* < 0.0011 for TGA vs TGAC, d: *p* < 0.0011 for TGA vs Col1, and e: *p* < 0.0011 for TGAC vs Col1). (B) COL1A1, FN1, HSP90AA1, HSP90AB1, ABCC1, and ABCG2 fold expressions in MCF-7 cells cultured in TGA, TGAC, Col1, or 2D and treated for 2 days with doxorubicin at 0.1 μM, after 14 days in culture for hydrogels and 7 days for 2D. 2^–ΔΔCt^ values of each sample were calculated with the ΔCts of cell-laden hydrogels or 2D cultures for 16 or 9 days, respectively. (C–E) MCF-7 viability after incubating the TGA (C), TGAC (D), and Col1 (E) nonbioprinted cell-laden hydrogels with two cell densities with doxorubicin for 48 h. (F) Fluorescence intensity of the negative controls (cell-laden hydrogels not treated with doxorubicin) used for the calculation of the cell viability in C–E).

Finally, we evaluated whether these bioprinted cell-laden hydrogels could be used for the screening of chemotherapy agents, by comparing the efficacy of doxorubicin. We selected doxorubicin as a drug model because it is widely used in chemotherapy against breast cancer.^67^ 2D culture was carried out to compare the efficacy of the drug with the bioprinted models, and they showed that doxorubicin was effective at doses below 0.1 μM (IC_50_ = 0.094 μM, Figure 10A). Then, 3D experiments were carried out. TDM cell-laded bioprinted hydrogels were cultured for 14 days to allow BCC proliferation, and then, they were treated with doxorubicin at different concentrations. The IC_50_ values obtained for the bioprinted samples were statistically significantly higher than the values achieved in 2D cultures (TGA: IC_50_ > 10 μM; TGAC: IC_50_ = 7.032 μM, Figure 10A). When treated with doxorubicin, the IC_50_ values were 70-fold higher than those of the 2D cultures. This reduction in chemotherapy agent sensitivity in 3D cultures vs 2D cultures has already been shown, with the cell–ECM and cell–cell interactions being important players in the differences observed.^68^ Surprisingly, TGAC bioinks showed lower IC_50_ than TGA bioinks. When comparing the MCF-7 viability of Col1 cell-laden hydrogels treated with doxorubicin, we observed that in three out of the four concentrations tested, TGA cell-laden hydrogels showed a statistically significant higher cell viability, whereas in TGAC hydrogels, only hydrogels treated with 0.1 μM had a statistically significant higher cell viability than Col1.

We hypothesized that the differences in drug response could be caused by the higher cell proliferation activity in the TGAC, making it more susceptible to doxorubicin.^69^ Therefore, to ensure that the differences in cell viability upon doxorubicin treatment were not caused by cell number differences among conditions, we prepared cell-laden hydrogels of TGA, TGAC, and Col1 having different cell densities (1.5 or 3 × 10^6^ cells/mL). Even with the high cell density difference between conditions (2-fold) on day 1, only TGA and TGAC had statistically significant different cell densities after 16 days in culture (Figure 10F). We found out that only at the highest doxorubicin concentration (100 μM) (Figure 10C–E), the cell density had an impact on drug efficacy in TGA and TGAC (Figure 10C,D), which did not explain the differences observed at concentrations below 100 μM. In an effort to clarify the differences observed between TGA and TGAC, we evaluated if there were any alterations in gene expression after the drug treatment. MCF-7-laden hydrogels were treated with doxorubicin at 0.1 μM for 48 h to ensure that the cell density was not the responsible for the differences observed in gene expression. We first evaluated the expression of tumor markers (HSP90AA1, HSP90AB1, COL1A1, and FN1). Cell-laden TGA hydrogels showed an upregulation of HSP90AB1 compared to TGAC, Col1, or 2D cultures. Conversely, there were no differences in gene expression of the tumor markers HSP90AA1, COL1A1, and FN1 between conditions, although the tendency to a lower expression of HSP90AA1 and a higher expression of FN1 in TGAC hydrogels vs TGA hydrogels was observed. We also evaluated the expression of ABCC1 and ABCG2, which have both been reported to be overexpressed in cancer cells resistant to doxorubicin.^70,71^ Again, no statistically significant differences were observed between conditions, implying that the differences observed in the drug response were not dependent on the expression of these MDR proteins. These findings suggest that the higher expression of HSP90AB1, an antiapoptotic protein, could be one of the reasons for the higher survival rate in the presence of doxorubicin. Indeed, we hypothesize that the presence of Col1 provokes a downregulation of HSP90AB1 in hydrogels made of TDMs with Col1, as in Col1 hydrogels, this gene is downregulated. Altogether, the addition of Col1 at 0.15%, although it improves the cell proliferation and printability, does not improve the biological properties of the TDM. However, modifications in Col1 concentration or the addition of other overexpressed proteins in breast tumors such as fibronectin could improve its biological properties. Overall, TGA bioinks have shown great potential to develop breast cancer models and to recreate the breast tumor microenvironment.

## 4 Conclusions

In this work, we have created a novel bioink formed by decellularized breast tissues to recreate the complex composition of breast tumors. We developed a method for obtaining decellularized porcine breast tissues rich in GAGs and collagen that meets the decellularization criteria previously established by other authors and that enables the formation of hydrogels. This material is biocompatible; however, the rheological properties were not suitable for its bioprinting. For this reason, a Pluronic bed and alginate at low concentrations (0.5–2%) were used. In order to support the TDM bioprinting without a bed, GelMA and alginate were added, having an optimal shape fidelity and printability, comparable with other proteinaceous hydrogels. We further tuned the bioink composition to be closer to the tumor ECM by adding Col1 so that it is overexpressed in breast tumors. The incorporation of Col1 improved the bioink printability and shape fidelity without affecting the Young’s modulus of the hydrogels, both being in the range of stiffness previously reported for breast tumors. To further analyze whether these bioinks will allow the fabrication of models with a precise location of cancer and stromal cells, we bioprinted constructs containing BCCs and hAMSCs. The construct consisted of an inner core of BCCs and an outer layer of stromal cells, which maintained its stability after bioprinting, with no cell and bioink blending. We then used the developed bioinks to print a breast tumor model. Both bioinks showed a high MCF-7 cell viability and high proliferation and allowed the formation of cell clusters or spheroids with a low expression of E-cadherin, a higher expression of tumor markers than in 2D cultures, and low chemotherapy responsiveness. The addition of Col1 increased cell proliferation as well as the drug sensitivity of the bioprinted tumors associated with a downregulation of HSP90AB1. Overall, these results demonstrate the great potential of these bioinks for fabricating breast tumor models by bioprinting. Further studies using these biomaterials will manifest the importance of recreating the complexity of the ECM using decellularized native tissues to ensure closer recreation of tumors to study the interactions of cancer and stromal cells and the extracellular matrix.

## Supporting Information Available

Additional experimental details; composition (%) and Young’s moduli (*E*) of the hydrogels; primers used in RT-qPCR; cell viability of MCF-7 cells in TGA and TGAC bioinks and in Col1 hydrogels; TDM bioprinting at different concentrations; MCF-7 viability in cell-laden TDM bioprinted hydrogels; temperature sweeps, bioprinting, and MCF-7 viability of TDM and alginate bioinks; MCF-7 viability with calcein AM/PI staining in TDM and alginate hydrogels; Young’s moduli of T2, T3, G2.5, and A0.5 hydrogels; amplitude sweeps and flow curves; cell proliferation in TGA and TGAC bioinks, Col1 hydrogels, and in 2D; CACNA1G and KCNA1Ct values (PDF)
