# Supplementary material for: Bioprinting Decellularized Breast Tissue for the Development of Three-Dimensional Breast Cancer Models
Source: ACS Appl Mater Interfaces. 2022 Jun 23;14(26):29467–82. doi: 10.1021/acsami.2c00920 (PMC9264314; doi:10.1021/acsami.2c00920)
Supplement: Supplementary file 1 — am2c00920_si_001.pdf [file am2c00920_si_001.pdf]

Supporting Information

Bioprinting Decellularized Breast Tissue for the Development of 3D Breast Cancer Models

Barbara Blanco-Fernandez, Sergi Rey-Vinolas, Gülsün Bağcı, Gerard Rubi-Sans, Jorge Otero, Daniel Navajas, Soledad Perez-Amodio, Elisabeth Engel**

EXPERIMENTAL SECTION

Collagen quantification. 100 µL of dry TDM resuspended in milliQ water or a standard of Collagen type 1 were incubated with 100 µL of HCl 12 M for 3 h at 120 ºC. Then, the water was evaporated at 60 ºC. Once evaporated, the pellet was resuspended in 100 µL of milliQ water, spin at 13,200 rpm 10 min and 30 µL of the supernatant of each sample were added to a 96-well plate. Then, 70 µL of a diluent (66.7% 2-propranolol in milliQ water) were added to each well. Afterwards, 50 µL of the oxidant solution was mixed and incubated for 5 min (Chloramine-T hydrate 1.573%, sodium acetate trihydrate 4.75%, sodium citrate tribasic dihydrate 3.56%, citric acid monohydrate 0.46%, 2-Propanol 33.32%, in milliQ water). Finally, 125 µL Color reagent was incorporated (4-(Dimethylamine) benzaldehyde 10.18%, perchloric acid 13.02%, 2-propanol 84.78%, in milliQ water), mixed and incubated at 70 ºC 15 min. The absorbance at 550 nm was recorded, and the amount of collagen was calculated with the corresponding calibration curve and normalized by the weight of TDM used.

SUPPLEMENTARY TABLES

Table S1. Composition (%) and young modulus (E) of the hydrogels tested in the mechanical properties.

Bioink	TDM	GelMA	Alginate	Col1	Irgacure	Cross-linking	E (kPa)
T2_G2.5_A0.5 (TGA)	2	2.5	0.5	-	0.1	37 ºC + $CaCl_2$ +UV	4.15 ± 0.66
T2_G2.5_A0.5_Col1 (TGAC)	2	2.5	0.5	0.15	0.1	37 ºC + $CaCl_2$+ UV	4.22 ± 1.02
T2	2	-	-	-	-	37 ºC	0.63 ± 0.15
T3	3	-	-	-	-	37 ºC	1.08 ± 0.24
A0.5	-	-	0.5	-	-	$CaCl_2$	2.96 ± 1.29
G2.5	-	2.5%	-	-	0.1	UV	0.95 ± 0.69
G2.5_A0.5	-	2.5%	0.5		0.1	37 ºC + $CaCl_2$	2.82 ± 0.52
G2.5_A0.5_Col1	-	2.5%	0.5	0.15	0.1	37 ºC + $CaCl_2$+ UV	2.15 ± 0.17
T2_A0.5	2	-	0.5	-	-	37 ºC + $CaCl_2$	3.67 ± 0.59
T2_A1	2	-	1	-	-	37 ºC + $CaCl_2$	5.97 ± 0.80

Table S2. Primers used in RT-qPCR.

Gene		***Sequence***
HSP90AA1	Forward	5'-TCCTGTGCGGTCACTTAGC -3'
	Reverse	5'-AAGGCGAACGTCTCAACCTC -3'
HSP90AB1	Forward	5'- GGGACTGTCTGGGTATCGGAA-3'
	Reverse	5'- AAGGCAAAAGTCTCCACCTCC-3'
ABCC1	Forward	5'-CCATCCACGACCCTAATCCC -3'
	Reverse	5'-ACTTGTTCCGACGTGTCCTC -3'
KCNA1	Forward	5'-AAAGACGACCTGCCGCATT-3'
	Reverse	5'-CCTTCTAAGCTCCCACCAGG-3'
CACNA1G	Forward	5'-CGAGATCAGCCTCCCACTTT-3'
	Reverse	5'-CTCAGCTAGAGGGATCTGTGGG-3'
ABCG2	Forward	5'-GACTTATGTTCCACGGGCCT -3'
	Reverse	5'-GGCTCTATGATCTCTGTGGCTTT-3'
FN1	Forward	5'-CCAGGCAGTACAATGTGGGT -3'
	Reverse	5'-TGGAATAGAGCTCCCAGGCT -3'
COL1A1	Forward	5'-GGAATGAAGGGACACAGAGGTT -3'
	Reverse	5'- AGTAGCACCATCATTTCCACGA-3'

Table S3. Cell viability of MCF-7 cells in bioprinted TGA and TGAC hydrogels and non-bioprinted Col1 hydrogels after 1 day in culture.

Hydrogel type	*Viability (%)*
TGA	60.02 ± 9.87
TGAC	65.51 ± 10.80
Col1	70.05 ± 8.16

SUPPLEMENTARY FIGURES

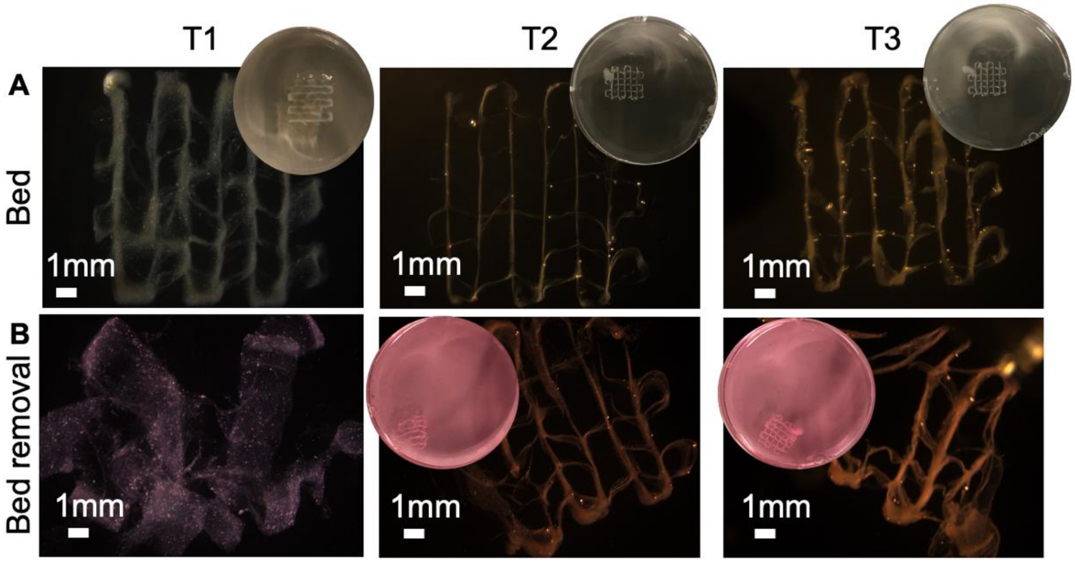

Figure S1. TDM bioprinting at different concentrations (10 mg/ml, 20 mg/mL and 30 mg/mL) containing 10^6 cells/mL of MCF-7 cells. (A) Cell-laden hydrogels bioprinted in a bed of Pluronic F127 at 23% (scale bar 1 mm). (B) Bioprinted cell-laden hydrogels after the bedding removal with several washes of cold PBS (scale bar 1 mm).

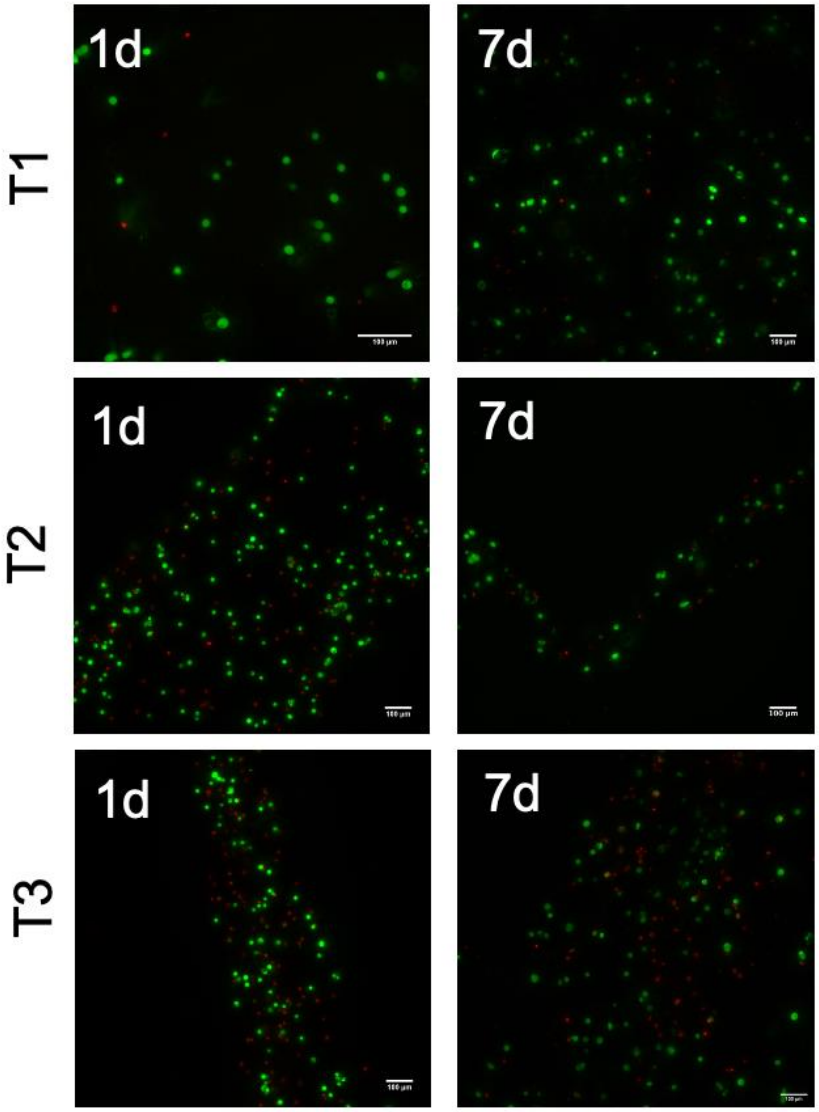

Figure S2. (A) MCF-7 viability in cell-laden TDM hydrogels bioprinted in the Pluronic F127 bed (10 mg/ml, 20 mg/mL and 30 mg/mL) and containing 10^6 cells/mL. Bioprinted hydrogels stained with a calcein AM/PI staining after 1 day or 7 days in culture. Viable cells in green and dead cells in red (scale bar: 100 μm).

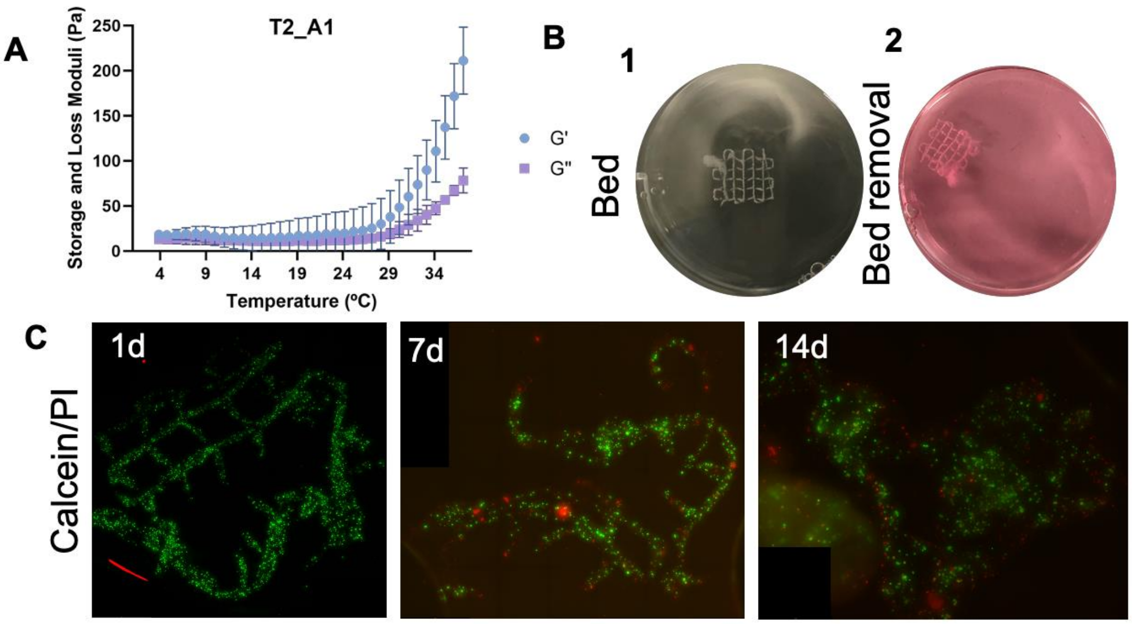

Figure S3. (A) Temperature sweep of bioink containing TDM at 20 mg/mL and alginate 1%. (B) Bioprinted MCF-7 laden scaffolds encapsulating 10^6 cells/mL. Hydrogels were bioprinted in a Pluronic F127 bed at 23% (1). Then, the bedding was removed with several washes of cold PBS (2). (C) MCF-7 viability over time in T2_A1 hydrogels. Cell-laden hydrogels were stained with a calcein AM/Propidium iodide (PI) staining. Viable cells in green and dead cells in red.

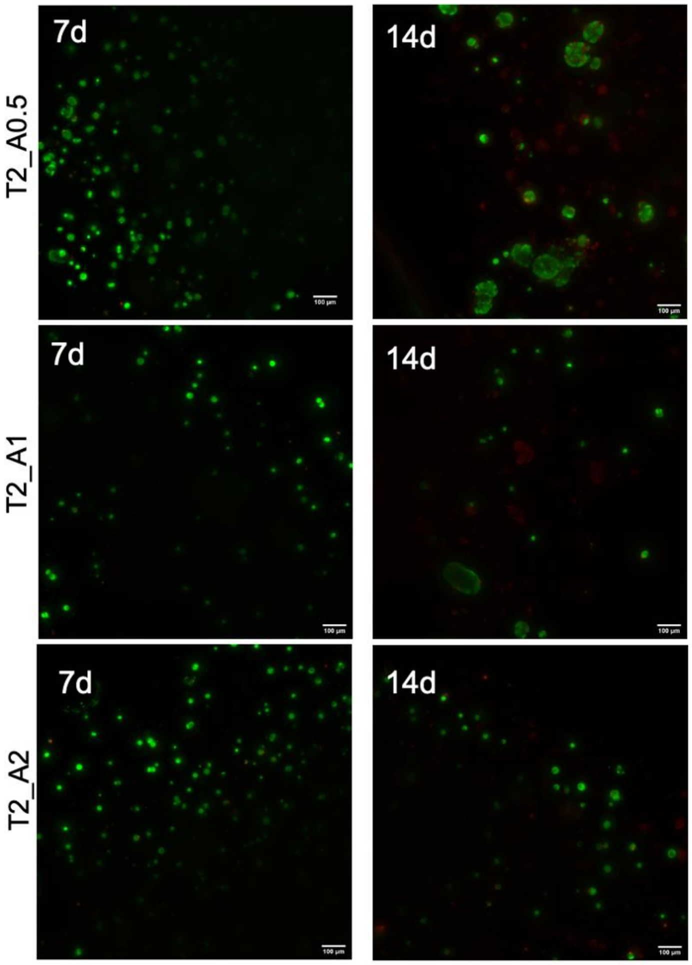

Figure S4. MCF-7 viability with a calcein AM/PI staining in hydrogels of TDM 20 mg/mL with alginate at 0.5%, 1% and 2% at day 7 and 14 (scale bar: 100 µm).

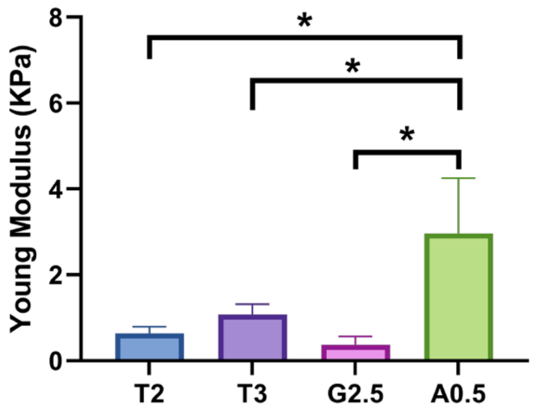

Figure S5. Young modulus of T2, T3, G2.5 and A0.5 hydrogels.

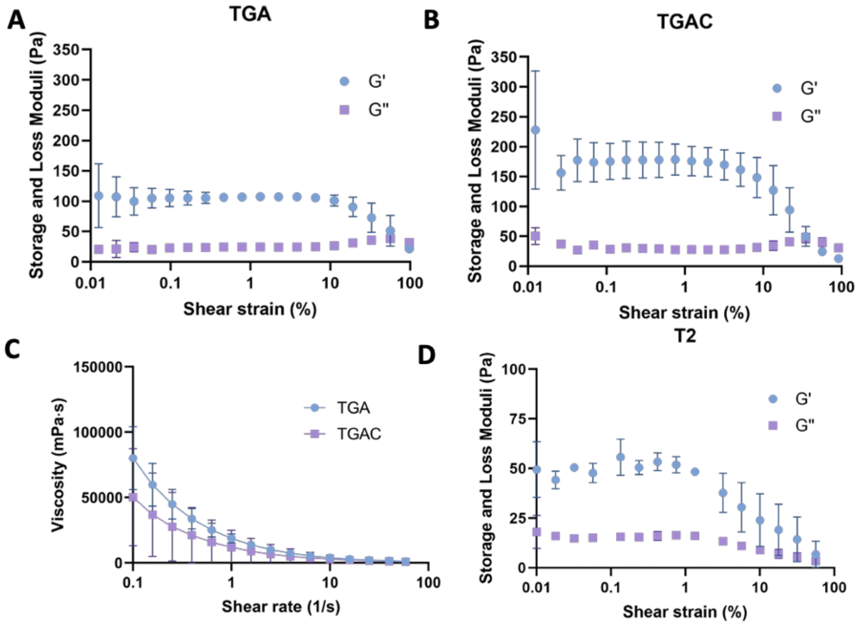

Figure S6. (A-B) Amplitude sweeps of TGA (A) and TGAC (B) at 8 ºC. (C) Flow curve of TGA and TGAC at 8 ºC. (D) Amplitude sweeps of T2 at 37 ºC.

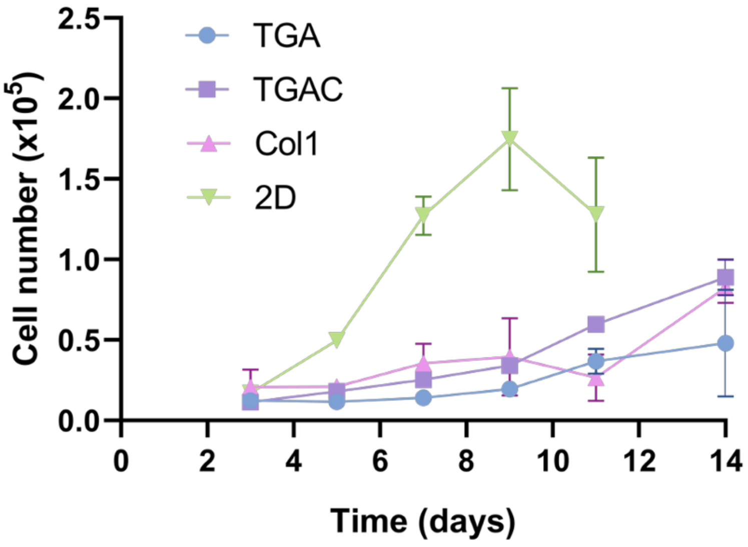

Figure S7. Cell proliferation, expressed in cell number, in the bioprinted TGA and TGAC hydrogels, non-bioprinted Col1 hydrogels and in 2D over time.

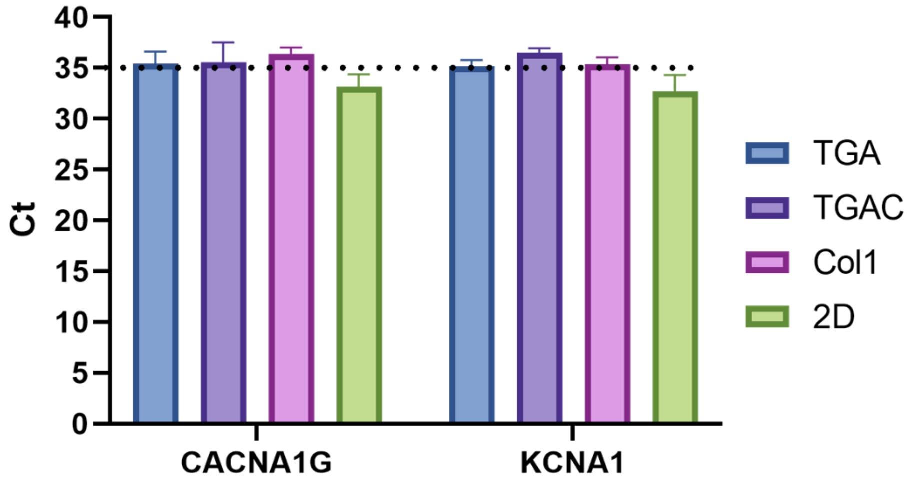

Figure S8. Ct values of CACNA1G and KCNA1 in MCF7 after 14 days in culture in TGA, TGAC and Col1 hydrogels or 9 days in 2D culture.
